# Experimental, Simulation and Theoretical Insights into Anisotropic Thermal Behavior of Epoxy Nanocomposites Reinforced with Carbonaceous Nanofillers

**DOI:** 10.3390/polym17091248

**Published:** 2025-05-03

**Authors:** Giovanni Spinelli, Rosella Guarini, Liberata Guadagno, Carlo Naddeo, Luigi Vertuccio, Vittorio Romano

**Affiliations:** 1 Faculty of Transport Sciences and Technologies, University of Study “Giustino Fortunato”, Via Raffaele Delcogliano 12, 82100 Benevento, Italy; 2 Open Laboratory on Experimental Micro and Nano Mechanics, Institute of Mechanics, Bulgarian Academy of Sciences, Acad. G. Bonchev Str., Block 4, 1113 Sofia, Bulgaria; rgrosagi@gmail.com; 3 Department of Industrial Engineering, University of Salerno, Via Giovanni Paolo II, 84084 Fisciano, Italy; cnaddeo@unisa.it (C.N.); vittorioromano2022@gmail.com (V.R.); 4 Department of Engineering, University of Campania “Luigi Vanvitelli”, Via Roma 29, 81031 Aversa, Italy; luigi.vertuccio@unicampania.it

**Keywords:** epoxy resin, thermal conductivity, simulation study, thermal anisotropy

## Abstract

Understanding and optimizing thermal conductivity in epoxy-based composites is crucial for efficient thermal management applications. This study investigates the anisotropic thermal conductivity of a tetra-functional epoxy resin filled with low concentrations (0.25–2.00 wt%) of carbonaceous nanofillers: 1D multiwall carbon nanotubes (MWCNTs) and 2D exfoliated graphite (EG) nanoparticles. Experimental measurements conducted using the Transient Plane Source (TPS) method reveal distinct behaviors depending on the nanofiller’s geometry. Epoxy formulations incorporating MWCNTs exhibit a ~60% increase in in-plane thermal conductivity (λ_I-p dir._) compared to the unfilled resin, with negligible changes in the through-plane direction (λ_T-p dir._). Conversely, EG nanoparticles enhance thermal conductivity in both directions, with a preference for the in-plane direction, achieving a ~250% increase at 2 wt%. In light of this, graphene-based fillers establish a predominant thermal transport direction in the resulting nanocomposites due to their layered structure, whereas MWCNTs create unidirectional thermal pathways. The TPS results were complemented by multiphysics simulations in COMSOL and theoretical studies based on the theory of thermal circuits to explain the observed phenomena and justify the experimental findings. This integrated approach, combining experiments, theoretical analyses, and simulations, demonstrates the potential for tailoring the thermal properties of epoxy nanocomposites. These insights provide a foundation for developing advanced materials optimized for efficient thermal management in high-performance systems.

## 1. Introduction

The efficient thermal management of heat dissipation is crucial in determining the performance, reliability, and longevity of advanced miniaturized devices used in various fields of technology [1]. These include, but are not limited to, electronic components such as central processing units (CPUs) and graphic processing units (GPUs) in computers, optoelectronic systems like high-power light-emitting diodes (LEDs), photonic devices, rechargeable batteries, and electronic packaging systems.

The miniaturization of devices and demand for higher performance increase power densities, generating more heat per unit volume. Without effective thermal management, overheating can degrade materials, reduce performance, and lead to device failure [2]. One promising approach to addressing this challenge is to enhance the thermal conductivity of the interface materials used within these systems. Thermal interface materials (TIMs) play a crucial role in efficiently transferring heat from the heat-generating components to the surrounding cooling systems [3]. Improving the thermal conductivity of TIMs ensures faster heat dissipation, minimizing thermal resistance at interfaces and mitigating the risk of thermal runaway. Also, studies, such as those by Razeeb et al. [4] and Pop et al. [5], have highlighted the importance of advanced TIMs for sustaining the reliability of next-generation electronics. Recent innovations in thermally conductive materials, especially graphene and CNTs, highlight their potential to enhance heat transfer in TIMs due to graphene’s high in-plane conductivity, and CNTs’ unidirectional thermal transport [6]. When incorporated into polymer matrices, such as epoxies, these nanomaterials form conductive networks that facilitate efficient heat dissipation across interfaces [7,8].

Improving the thermal conductivity of TIMs is essential for effective heat management, preventing overheating, and extending the lifespan of a device in advanced electronic, optoelectronic, and photonic technologies. Polymer-based TIMs are widely used due to their advantageous properties, including their lightweight nature, making them ideal for aerospace and portable applications. They are also easy to process, allowing for versatile fabrication, and offer excellent corrosion resistance, ensuring durability in harsh environments. Additionally, their cost-effectiveness compared to metal or ceramic alternatives makes them highly suitable for large-scale manufacturing and commercial use.

However, a significant drawback of conventional polymers is their inherent thermal insulation. Most unmodified polymers have a low thermal conductivity, typically ranging from 0.1 to 0.5 W/m·K, which is orders of magnitude lower than metals (e.g., copper: ~400 W/m·K, aluminum: ~200 W/m·K) or advanced carbon-based materials (e.g., graphene: >2000 W/m·K) [9]. The low thermal conductivity of polymers limits their effectiveness in applications requiring efficient heat dissipation. To overcome this, researchers have developed strategies to enhance their conductivity while preserving their advantages. A widely used method is the incorporation of thermally conductive fillers, such as ceramic particles (e.g., aluminum oxide, boron nitride) or carbon-based materials (e.g., graphene, carbon nanotubes), which form thermal pathways within the polymer matrix. Notably, graphene nanoplatelets, with their exceptionally high thermal conductivity, can significantly improve polymer composites’ heat transfer while maintaining their lightweight and flexible properties [10].

Similarly, boron nitride fillers have demonstrated effective thermal enhancement, leveraging their anisotropic heat transport properties and chemical stability [11]. Despite these advancements, the performance of TIMs is often constrained by factors such as the dispersion quality of the fillers, interfacial thermal resistance between the filler and the polymer matrix, and the percolation threshold required to form a continuous thermal network [12]. Enhancing polymer-based TIMs through surface functionalization or optimized filler alignment remains crucial for improving thermal performance. Recently, CNTs and graphene nanoparticles have gained attention for their nanostructures and exceptional properties, including high mechanical strength, electrical conductivity, and superior thermal conductivities of up to 3000 W/m·K and 5000 W/m·K, respectively [13,14]. However, the thermal conductivity (λ) of carbon-based composites falls significantly short of the values estimated from theoretical predictions [15,16,17].

This discrepancy stems from challenges in achieving uniform graphene dispersion, strong interfacial adhesion, and optimal orientation within composites. Extensive research has focused on improving filler dispersion and interfacial properties, resulting in significant advancements [18,19,20]. Graphene exhibits strong intrinsic anisotropy, primarily due to its unique two-dimensional (2D) structure composed of sp^2^-hybridized carbon atoms arranged in a hexagonal lattice along the basal plane [21]. This structural configuration results in highly directional thermal transport behavior. Experimental studies have demonstrated that graphene’s in-plane thermal conductivity is remarkably high, ranging between 1000 and 5300 W/m·K, whereas its cross-plane (or through-plane) thermal conductivity is significantly lower, typically falling within the range of 5 to 20 W/m·K [22]. This stark contrast in thermal conductivity arises from the strong covalent bonds within graphene’s basal plane, enabling efficient phonon transport, while weak van der Waals forces between layers create thermal resistance in the through-plane direction. Similarly, carbon nanotubes (CNTs) exhibit high axial thermal conductivity (>3000 W/m·K) but lower radial conductivity due to weak inter-tube interactions. Unlike graphene’s planar heat flow, CNTs’ quasi-1D structure directs heat along their length, with additional phonon scattering from their curvature. These differences are key to choosing between graphene and CNTs for thermal management, depending on heat dissipation needs and integration strategies.

A novel approach for estimating the overall thermal conductivity of particulate composites with interfacial thermal resistance is presented by Nan et al. [23]. This method integrates an effective medium theory with the fundamental principle of Kapitza thermal contact resistance to provide a comprehensive predictive framework. Regarding the aforementioned approach, Chu et al. conducted an initial and interesting investigation into the controlled orientation of graphene within a metal matrix and its influence on the directional thermal properties of the resulting composites [24]. Their findings revealed an inverse anisotropic relationship between thermal conductivity (TC) and the coefficient of thermal expansion (CTE) with increasing graphene nanoplatelet (GNS) content. Notably, in-plane TC showed significant enhancement over through-plane TC, emphasizing the importance of graphene alignment in heat dissipation.

The directional dependence of thermal characteristics in boron carbide–graphene platelet composites was observed by Rutkowski et al. [25]. Chen and Gao investigated the transport behavior of graphene-nanoplatelet-based composite materials to identify the origins of anisotropy and explore effective methods for its control [26]. Currently, numerous studies have explored the anisotropic heat transfer properties of carbon-based materials [27,28]. However, there is a lack of comprehensive reviews addressing their development, existing challenges, and future perspectives.

This work experimentally investigates the intrinsic anisotropic thermal transport that takes place in nanocomposites incorporating one-dimensional CNTs and two-dimensional (exfoliated graphite, EG) fillers. To support the experimental findings, a morphological analysis is conducted to assess the dispersion of fillers within the epoxy matrix, which serves as the composite phase. Furthermore, multiphysics simulations are performed to gain deeper insights and, more importantly, to provide a three-dimensional graphical analysis of the thermal properties of these materials.

Additionally, a theoretical study based on an equivalent thermal circuit model is presented, focusing on graphene-based composites, where thermal anisotropy is more pronounced compared to CNT-based systems.

This study represents a significant advancement in understanding the anisotropic thermal conductivity of epoxy-based nanocomposites, which is a phenomenon that has already been experimentally observed and reported in the literature. It introduces a novel approach by integrating experimental measurements, theoretical analyses, and multiphysics simulations. To the best of our knowledge, this is one of the first studies to combine these methodologies to explain the thermal behavior of carbon-based composites. The results demonstrate that the anisotropy in thermal conductivity is more pronounced in graphene-based composites compared to carbon nanotube (MWCNT)-based composites. Specifically, graphene significantly enhances thermal conductivity in both the in-plane and through-plane directions, with a preference for the in-plane direction. In contrast, MWCNTs primarily enhance in-plane conductivity with little effect on the through-plane direction. Given the more pronounced anisotropic behavior in the graphene-based composites, the numerical simulations and theoretical modeling were focused specifically on this type of filler, providing deeper insights into the mechanisms driving the observed thermal transport properties. The use of COMSOL (version 6.1) simulations and theoretical modeling based on the thermal circuit theory provides a comprehensive framework for interpreting the experimental results, offering new insights into the role of nanofiller and its dispersion within the matrix on the overall thermal transport properties of the resulting structure. By employing this integrated approach, this study not only enhances our understanding of the thermal properties of epoxy nanocomposites but also presents a predictive tool for tailoring these properties to optimize thermal management in high-performance systems.

This work contributes significantly to the field of polymers and paves the way for the development of advanced materials designed for efficient thermal dissipation. In fact, the work is motivated by the increasing demand for efficient thermal management in electronic and structural applications, where traditional epoxy resins show limited thermal performance. The addition of carbon-based nanofillers offers a promising route to enhance thermal conductivity despite the fact that a clear understanding of anisotropic heat transport mechanisms remains incomplete. By integrating experimental measurements with theoretical and numerical modeling, this study aims to clarify these mechanisms and support the design of advanced nanocomposites with tailored thermal properties.

## 2. Materials and Methods

### 2.1. Sample Preparation

The epoxy matrix was formulated by blending tetraglycidyl methylene dianiline (TGMDA), an epoxy precursor with an equivalent weight of 117–133 g/eq, with 1,4-butanediol diglycidyl ether (BDE), a reactive monomer serving as a diluent. The curing process was facilitated by the addition of 4,4′-diaminodiphenyl sulfone (DDS) as the hardener. All chemicals were provided from Sigma-Aldrich (Milan, Italy).The epoxy system was prepared by maintaining an 80:20 TGMDA-to-BDE weight ratio, optimizing the balance between crosslinking density and matrix flexibility. The incorporation of BDE as a reactive diluent in unfilled and nanofilled epoxy formulations based on TGMDA has effectively reduced viscosity, enhancing processability and filler dispersion within the polymer network [29]. For brevity, hereafter, the unfilled epoxy resin is denoted as TBD (TGMDA + BDE + DDS), while the nanocomposites are labeled as TBD+X(nanofiller), where X represents the nanofiller percentage.

**Figure 1:**
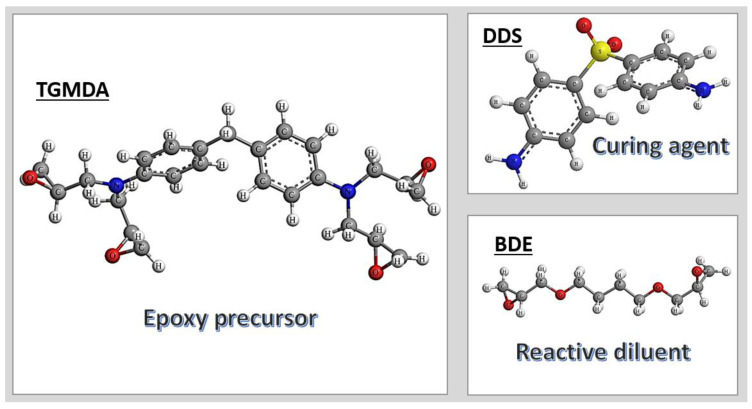
Chemical structures of compounds used for the TBD epoxy matrix nanocomposites.

Figure 1 illustrates the chemical structures of the compounds utilized in the TBD epoxy matrix nanocomposites.

Carbon-based nanostructures, specifically multiwall carbon nanotubes (MWCNTs) and exfoliated graphite (EG) were incorporated into an epoxy resin matrix to enhance its physical properties. The MWCNTs (Grade 3100) were sourced from Nanocyl S.A. (Sambreville, Belgium), and Transmission Electron Microscopy TEM (TEM-JEOL model JEM-1400 Plus-Akishima, Tokyo, Japan) analysis revealed that the outer diameter of the MWCNTs ranged from 10 to 30 nm, with lengths varying from hundreds of nanometers to several micrometers. These nanotubes exhibited wall counts ranging from 4 to 20 layers [30]. The specific surface area of the MWCNTs, as determined using the Brunauer–Emmett–Teller (BET) method, was approximately 250–300 m^2^/g. The carbon purity of the MWCNTs was >95%, with metal oxide impurities constituting less than 5%, as confirmed by thermogravimetric analysis [30].

Exfoliated graphite (EG) was prepared from natural flake graphite provided by Superior Graphite (Chicago, Illinois, USA). It is characterized by the following parameters: carbon purity % = 98.6; size: 1” × 8 mesh; bulk density: (g/100 mL) = 17.80; and sulfur (%) = 0.056. Starting with natural graphite with an average diameter of 500 μm, the EG was prepared as follows: A mixture containing nitric and sulphuric acid (volume ratio = 135 mL/255 mL) and natural graphite was prepared. After 24 h of reaction, intercalation within graphene sheets took place to form intercalated graphite. Then, the mixture was filtered, washed with water, and dried in an oven at low temperatures. The intercalated graphite compound was subjected to a sudden heat treatment temperature of 900 °C, and rapid expansion then occurred.

The epoxy resin blend and DDS (diamino-diphenyl sulfone) were mixed at 120 °C, followed by the addition of the carbon-based nanoparticles, which were integrated into the matrix using ultrasonic processing for 20 min. The resulting epoxy nanocomposites were prepared with two different nanoparticle loadings, specifically 0.5% and 1% by weight. To perform thermal analysis measurements, silicone molds with suitable geometry and dimensions were prepared to obtain cylindrical samples with a thickness of 10 mm and a diameter of 50 mm. After sonication, the fluid epoxy mixture loaded with EG was slowly poured into the silicone mold. Then, the samples were solidified through a curing process consisting of a two-stage isothermal cycle: an initial stage at 125 °C for 1 h and a second stage at 200 °C for 3 h.

### 2.2. Thermal Analysis

The study measured anisotropic thermal conductivity using the Hot Disk^®^ 2500S thermal analyzer (mod 2500S, Hot-Disk AB, Gothenburg, Sweden), following the ISO 22007-2 standard [31]. The Transient Plane Source (TPS) method was applied to evaluate the in-plane (λ_I-p dir._) and through-plane (λ_T-p dir._) thermal conductivity of carbon nanotube (CNT) and exfoliated graphite (EG) epoxy-based nanocomposites, which were assessed along the x- and z-axis, respectively. A nickel sensor was placed between the cylindrical sample pieces, acting as a heat source and temperature sensor. A current pulse generated heat, and the increase in temperature over time was recorded via the change in electrical resistance (R(t)).

The sensor’s resistance change is expressed as follows:

(1) $$R\left(t\right)=R_{0}\left[1+\alpha \Delta T_{i}+\alpha \Delta T\left(\tau \right)\right]$$

where R_0_ is the initial resistance of the sensor before heating [Ω], ΔT_i_ is the temperature difference across the heat sensor insulation layer [K], and ΔT(τ) represents the temperature increase in the sensor’s outer surface [K]. Naturally, the temperature coefficient of the electrical resistance of the heating element (α, [1/K]) must be known. The mean increase in temperature in the material is given by the following equation:

(2) $$∆T=\frac{P_{0}}{f\left(\lambda_{I-p\;dir.},\lambda_{T-p\;dir.}\right)}D\left(\tau \right)$$

where P_0_ is the constant heating power of the sensor [W], f(λ_I-p dir._, λ_T-p dir._) is a function of uniform in-plane and trough-plane thermal conductivity [W/m K] and $D\left(\tau \right)$ is a dimensionless time function.

Further equations to determine the thermal properties are the following:

(3) $$\lambda_{I-p\;dir.}=\alpha_{I-p\;dir.}\cdot C;\;\lambda_{T-p\;dir.}=\alpha_{T-p\;dir.}\cdot C$$

where $\alpha_{I-p\;dir.}$ and $\alpha_{T-p\;dir.}$ are the measured thermal diffusivity values, C = ρ∙c_p_ is the heat capacity per volume [J/(m^3^s)] calculated in advance, using a Differential Scanning Calorimeter according to the ASTM E1269/DIN 51007 [32], and ρ and c_p_ are the density [kgm^−3^] and the specific heat [J kg^−1^K^−1^].

Five measurements per sample were averaged for reliability, omitting the initial 50 data points to mitigate the influence of contact resistance. The probing depth and optimal measurement time (80 s at 0.1 W) were determined to ensure accurate results while minimizing sensor–sample contact resistance effects.

**Figure 2:**
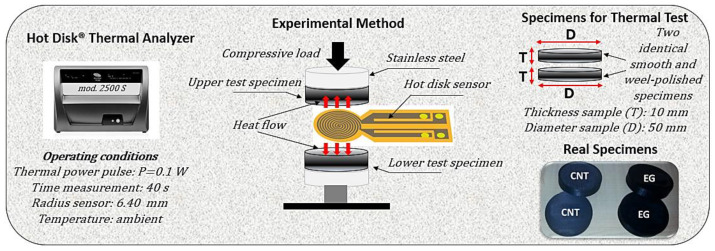
The Hot Disk^®^ 2500S thermal analyzer, accompanied by a schematic representation of the TPS sensor and actual specimens, including a diagram illustrating their geometrical characteristics.

This method precisely characterizes thermal transport properties in anisotropic materials, which are essential for nanocomposite applications. Spinelli et al. [33] extensively documented the complete mathematical framework and methodology employed in the TPS technique. Figure 2 provides a visual overview highlighting the key principles of this approach for reference.

### 2.3. Morphological Analysis

**Figure 3:**
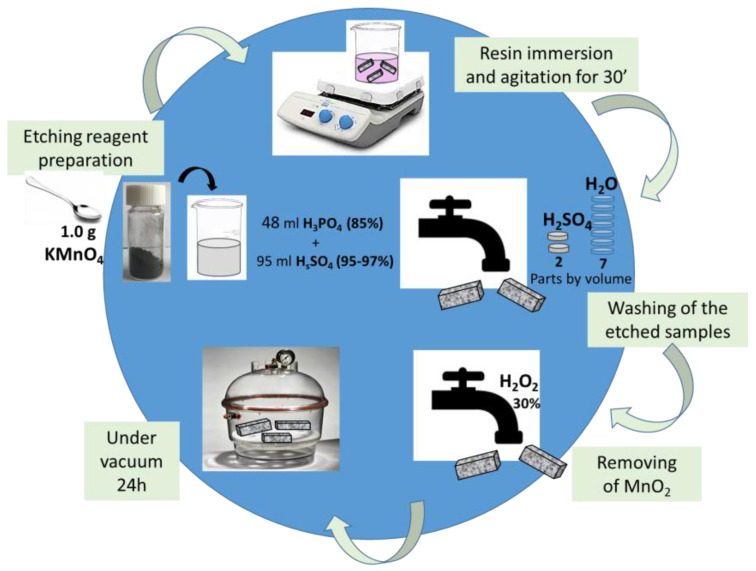
Schematic representation of the etching procedure.

The morphological characteristics of the obtained nanocomposites were analyzed using a Field Emission Scanning Electron Microscope (FESEM) (JSM-6700F, JEOL- Akishima, Tokyo, Japan), operating at 3 kV. Thin sections of the nanocomposites were prepared by slicing solid samples with a sledge microtome, followed by an etching process before the FESEM observation, as schematically summarized in Figure 3. The etching solution was prepared by dissolving 1.0 g of potassium permanganate in a mixture of 95 mL sulfuric acid (95–97%) and 48 mL orthophosphoric acid (85%) under continuous stirring. The resin-filled samples were submerged in this freshly prepared reagent at room temperature, with agitation maintained for 36 h. To remove residual reagents, the samples underwent a multi-step washing process: first, they were rinsed with a cold solution composed of 2 parts concentrated sulfuric acid and 7 parts water. This was followed by treatment with a 30% hydrogen peroxide aqueous solution to eliminate any remaining manganese dioxide. Finally, the samples were washed thoroughly with distilled water and left under a vacuum for 5 days to ensure complete drying. The etching procedure adopted in this work has proved very effective in analyzing the correlation between the nanofiller properties and related composites [34].

### 2.4. Multiphisics Study

Experimental findings on thermal anisotropy were extensively analyzed using COMSOL Multiphysics^®^ (version 6.1) simulations based on the Finite Element Method (FEM). The approach uses representative volume elements (RVEs) with different numbers of filler particles to capture thermal transport accurately. This study focuses on graphene-based composites, which show more pronounced anisotropic behavior than CNT-filled systems, with the Heat Transfer in Solids module used for thermal simulations. The simulations are based on applying a temperature difference in two distinct directions: (i) in the through-plane, along the thickness of the composite, and (ii) in-plane, aligned with the graphene sheet orientation. Given the inherent symmetry of the structure, the thermal behavior along the y-direction mirrors that which is observed in the x-direction. As a result, the corresponding data and analysis for the y-direction do not provide additional insights and have been intentionally omitted throughout the discussion to maintain clarity and conciseness. This choice allows for a more streamlined presentation without compromising the completeness of the study.

**Figure 4:**
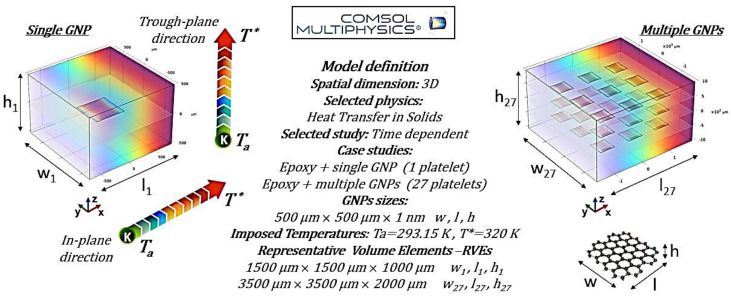
Essential model definitions for the numerical analysis and schematic representation of the case studies examined in the present work.

Figure 4 depicts the essential model definitions used in the simulations alongside a schematic representation of the case studies considered.

This dual line of investigation allows for a detailed evaluation of key thermal properties, particularly heat flux and temperature distribution, as well as effective thermal conductivity in different orientations.

A method for selecting the representative volume element (RVE) in COMSOL simulations was specifically developed to isolate and analyze the thermal transport arising solely from the dispersed filler. Once the geometry of the graphene nanoplatelet was defined, both in the case of a single particle and a system of 27 particles, the chosen RVE size preserved equivalent alternative thermal pathways for both the filler and the matrix in the x and z directions. Importantly, the consistency of the results between the simplified and more complex configurations demonstrates that the approach is valid and not inherently dependent on particle numbers. Consequently, it can be extended to model any filler concentration, with the only limiting factor being computational cost.

The governing heat balance equations are explicitly reported below, while the initial and boundary conditions applied in both simulation cases, ensuring a well-posed problem, are summarized in Table 1.

In detail, the thermal energy equation governing the heat conduction in a solid (at constant pressure) can be written as a three-dimensional Cartesian coordinate system, considering a small finite differential volume element characterized by the dimensions *dx*, *dy*, and *dz* along the x-, y-, and z-axes, respectively, as shown in Equation (4) for graphene and Equation (5) for the polymer.

(4) $$\lambda_{gx,y}\left[\frac{\partial^{2}T}{\partial x^{2}}+\frac{\partial^{2}T}{\partial y^{2}}\right]+\lambda_{gz}\frac{\partial^{2}T}{\partial z^{2}}=\rho_{g}c_{pg}\frac{\partial T}{\partial t}$$

(5) $$\lambda_{m}\left[\frac{\partial^{2}T}{\partial x^{2}}+\frac{\partial^{2}T}{\partial y^{2}}+\frac{\partial^{2}T}{\partial z^{2}}\right]=\rho_{m}c_{pm}\frac{\partial T}{\partial t}$$

where ρ_g_ and ρ_m_ are the graphene and polymer density [kgm*^−^*^3^], respectively; c_pg_ and c_pm_ represent their specific heat [J kg*^−^*^1^K*^−^*^1^]; and λ_m_, λ_gx,y_ and λ_gz_ respectively represent the thermal conductivity of the polymeric material, the thermal conductivity of graphene in the x- and y-directions (which are equal), and the thermal conductivity of graphene in the z-direction, perpendicular to its plane, all expressed in [Wm*^−^*^1^K*^−^*^1^].

Finally, $\frac{\partial^{2}T}{\partial x^{2}}$,$\;\;\frac{\partial^{2}T}{\partial y^{2}},\;\;\frac{\partial^{2}T}{\partial z^{2}}$ represent the second-order partial derivatives of the temperature function T with respect to the spatial coordinates *x*, y, and *z*.

### 2.5. Thermal Circuit Theory

The thermal behavior of the composite material was analyzed along both the in-plane and through-plane directions using the thermal circuit theory. This approach enables a simplified yet effective representation of heat transfer mechanisms by modeling the composite as an equivalent network of thermal resistances. By applying this method, it was possible to capture the anisotropic heat conduction behavior of graphene-based composites, supporting both numerical simulations and experimental findings.

**Figure 5:**
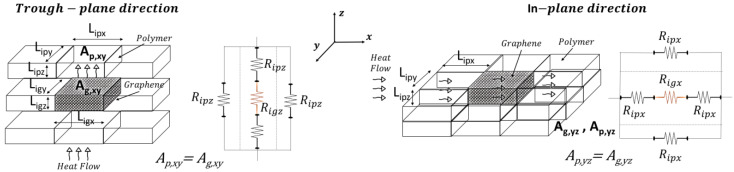
Schematic representation of the thermal circuit theory applied to the graphene-based nanocomposite in the trough-plane direction (**left**) and in-plane direction (**right**).

Similarly to the simulation study, the theoretical calculations assume the same symmetry condition, whereby the results along the y-direction are equivalent to those along the x-direction. Therefore, they are not reported, as they would be redundant and offer no additional insight. As illustrated in Figure 5, the through-plane and in-plane heat transport (the left and right schematic, respectively) was modeled as a parallel and series arrangement of thermal resistances, the parameters of which are dictated by the dimensions of the polymer and graphene subdomains into which the representative domain of the composite is divided as well as by the intrinsic thermal conductivities of both the polymer matrix and the graphene filler in its two directions.

In detail, heat flows sequentially through alternating polymer and graphene layers or exclusively polymer layers, with each material contributing a resistance term (R_ip_ and R_ig_).

Regarding the heat transfer through the plane (j = z) or in the plane (j = x), the total thermal resistance in the direction of flow (R_j_) is determined by the resistance in parallel in the plane orthogonal to the direction of flux and by the resistance in series along the direction of flux with which each parallel resistance is composed; the resistance offered by the individual subdomain layers of graphene and polymer is defined as follows:

(6) $${\left(R_{ig}\right)}_{j}=\frac{L_{igj}}{\lambda_{g,j}\cdot A_{g⊥j}}and{\left(R_{ip}\right)}_{j}=\frac{L_{ipj}}{\lambda_{p}\cdot A_{p⊥j}}$$

where L_ipj_ and L_igj_ are the thicknesses of the polymer and graphene layers, respectively; λ_p_ and λ_g,j_ denote the thermal conductivities of the polymer and that of graphene in the j-direction, and $A_{p⊥j}=A_{g⊥j}$ represents the cross-sectional area of heat transfer, expressed as follows:

(7) $$A_{p⊥j}=A_{p,yz}=L_{ipy}\cdot L_{ipz}\;for\;j=x\;and\;A_{p⊥j}=A_{p,xy}=L_{ipx}\cdot L_{ipy}\;for\;j=z$$

(8) $$A_{g⊥j}=A_{g,yz}=L_{igy}\cdot L_{igz}\;for\;j=x\;and\;A_{g⊥j}=A_{g,xy}=L_{igx}\cdot L_{igy}\;for\;j=z$$

In the above equation, L_ipy_, L_ipx_, and L_ipz_ represent the length, width, and thickness of the polymer domain along the x-, y-, and z-axes, respectively, while L_igy_, L_igx_, and L_igz_ denote the corresponding dimensions of the graphene domain.

The thermal circuit approach enables the determination of the heat flow components in both directions using the following governing equation:

(9) $$Q_{j}=\frac{1}{R_{j}}\cdot {∆T}_{j}$$

where Q_j_, Rj, and ΔT_j_ represent the heat flow, the overall resistance, and the corresponding imposed temperature differences in the through-plane and in-plane directions. Once these quantities are known, the corresponding thermal conductivities can be determined using Fourier’s law of heat conduction, according to which the conduction of heat flux in one direction is proportional to the temperature gradient in the same direction as the constant of proportionality coincides with the thermal conductivity; this is shown by the following equation:

(10) $$q_{j}=-\lambda_{j}\cdot \frac{dT_{j}}{dj}\;with\;j=z\;or\;x$$

where q_j_ is the heat flux (the amount of heat flowing per unit area per unit of time in units of W/m^2^) in the investigated directions (z and x), λ_j_ is the thermal conductivity of the material (in units of W/mK), and dT_j_/d_j_ is the temperature gradient in the direction of heat transfer (in units of K/m).

The analysis was conducted with reference to a representative volume containing a single graphene platelet oriented parallel to the *xy*-plane. This setup allows for an isolated evaluation of the influence of the filler on the effective thermal conductivity of the resulting composite when the temperature difference is applied along the x or z direction. This approach can be systematically extended, with appropriate considerations, to a larger number of filler particles (27 in this study), even when arranged arbitrarily. The latter case has been addressed using multiphysics simulation software to optimize computational feasibility. In all cases, the selected geometry was specifically designed to ensure identical spacing for the resin and graphene in the axial (z-direction) and in-plane (x-direction) directions. This configuration allows for an exclusive focus on the effect of heat flux propagation along these two dimensions while eliminating geometric factors that could otherwise influence the results. For instance, in the simplest case of a single graphene sheet, which serves as the initial reference due to its fundamental nature, moving along either the z-direction or the x-direction always results in encountering identical resin/graphene subdomain dimensions. This ensures a uniform and controlled environment for analyzing the heat flux behavior without introducing additional complexities related to geometric variations. In summary, this theoretical framework aims to quantify the impact of graphene’s anisotropic thermal properties on heat conduction, providing fundamental insights into the design of thermally efficient nanocomposites. This analytical treatment is a valuable tool for validating the experimental measurements and the numerical simulations performed in this study. Combining the thermal circuit approach with finite element simulations performed in COMSOL Multiphysics makes it possible to accurately quantify the anisotropic heat transport properties and predict effective thermal conductivity in both directions.

## 3. Results and Discussions

This section presents experimental results on the thermal conductivity of carbon-based composites at different weight percentages. A detailed morphological analysis was conducted to examine the dispersion and arrangement of the fillers within the polymer matrix. To further support the experimental findings, a multiphysics simulation study was performed, providing a comprehensive understanding of the thermal performance of the composites. A theoretical study based on a thermal equivalent circuit was also introduced to model heat transfer behavior.

In detail, in our study, the theoretical results refer to the outcomes derived from analytical models based on established physical principles and mathematical formulations. These models provide a simplified yet insightful understanding of the system under idealized assumptions. On the other hand, the simulation results were obtained through numerical methods that took into account more complex parameters and boundary conditions. A key advantage of the simulation approach is that it allows for a three-dimensional graphical inspection of the system’s behavior, which is not feasible through purely theoretical models. This visual and spatial representation provides a deeper understanding of the material’s response and structure, making it a major distinction between the two approaches. Despite these differences, the agreement between theoretical predictions and simulation outcomes validates the reliability of both approaches and supports the robustness of our conclusions. In brief, the combination of experimental, theoretical, and numerical approaches allows for a thorough evaluation of the key factors influencing thermal conductivity, ensuring a comprehensive understanding of the system under investigation.

### 3.1. Experimental Thermal Conductivity Evaluation

**Figure 6:**
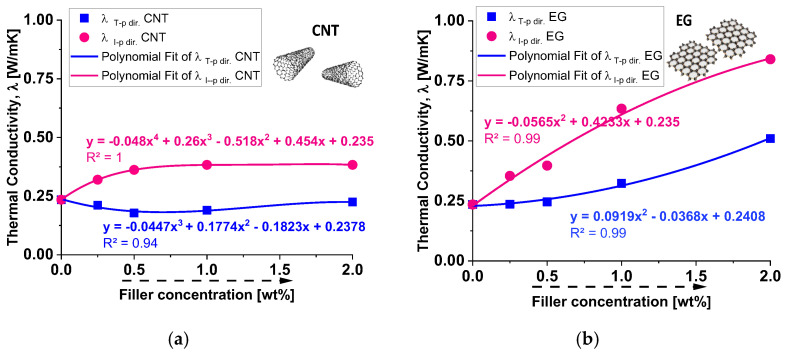
In-plane (λ_I-p dir._) and trough-plane (λ_T-p dir._) thermal conductivities of epoxy resin filled with different concentrations of carbon nanotubes CNTs (**a**) and exfoliated graphite EG (**b**).

Figure 6 compares the experimental results on the thermal conductivity (λ) of composites of tetrafunctional epoxy resin, modified with low weight percentages (up to 2% wt) of fillers: carbon nanotubes (CNTs) are represented on panel (a) and exfoliated graphite (EG) is shown on panel (b). The thermal conductivity is analyzed in two directions: the through-plane (λ_T−p dir._, represented by blue squares) and in-plane (λ_I-p dir._, represented by pink circles) directions. The data clearly indicate that, in accordance with the current literature [35,36], epoxy composites incorporating 2D-exfoliated graphite nanoparticles exhibit superior heat transport properties compared to those loaded with one-dimensional carbon nanotubes (CNTs). This observation underscores the improved thermal conductivity of epoxy composites enhanced by exfoliated graphite, highlighting the distinct influence of nanoparticle morphology on heat conduction.

As already reported in the literature [37], the transport of thermal energy in carbon-based nanostructures primarily occurs through a phonon conduction mechanism, which is influenced by factors such as the number of active phonon modes, boundary surface scattering, the free path length of electrons and phonons, and inelastic Umklapp scattering. In multiwalled carbon nanotubes (MWCNTs), the 1D tubular structure tends to form entangled networks, and the epoxy resin may not effectively wet the inner surfaces. This poor interfacial contact results in a high thermal boundary resistance, also known as Kapitza resistance (*R*_k_), which limits heat transfer between the filler and the organic matrix. Conversely, exfoliated graphite (EG), with its 2D planar structure, provides a significantly larger interfacial contact area with the epoxy matrix. The easier wetting of the nanofiller surfaces enhances the adhesion of graphene sheets to the matrix, forming a well-connected thermal network within the composite. This arrangement is ideal for efficient phonon transport, leading to a much lower Kapitza resistance than CNTs and, consequently, improved thermal conductivity.

In detail, the experimental results of Figure 4 also show that for CNT-based composites, the through-plane conductivity (λ_T−p dir.)_ remains relatively low, with a slight increase as the filler concentration increases. The in-plane conductivity (λ_I−p dir._) initially increases but then stabilizes at around 0.4 W/mK. Overall, CNTs provide moderate enhancement in in-plane thermal conductivity but have a limited impact on through-plane conductivity. For EG-based composites, the through-plane conductivity (λ_T−p dir._) increases more significantly than in CNT-based composites, suggesting better heat transfer in this direction. The in-plane conductivity (λ_I−p dir._) follows a strong upward trend, reaching values close to 1 W/mK at a 2 wt% filler concentration. Compared to CNTs, EG demonstrates superior enhancement in both thermal conductivity directions, especially in the in-plane direction. To summarize, EG-based composites outperform CNT-based composites in both in-plane and through-plane conductivity. CNTs exhibit weaker improvements in through-plane conductivity, likely due to their elongated shape and orientation within the matrix. EG provides a more efficient heat transfer network, especially in the in-plane direction, due to its layered structure and higher intrinsic thermal conductivity. These results suggest that EG would be a more effective filler for applications requiring high thermal conductivity than CNTs, particularly for enhancing in-plane heat dissipation. To conclude, as a result of the experimental analysis, a filler concentration of 2 wt% proved to be the most effective in enhancing thermal conductivity, regardless of the filler type. However, the graphene-based composite significantly outperformed the carbon nanotube composite, confirming its superior thermal efficiency. Furthermore, the comparison of thermal conductivity results shows distinct behaviors for the two types of fillers. For CNT-based composites, thermal conductivity increases with filler concentrations up to a certain point, after which it reaches a plateau, likely due to agglomeration and reduced efficiency in the heat transfer network. In contrast, for graphene-based composites, thermal conductivity continues to increase across the explored concentration range, indicating that graphene enhances the heat transfer process more effectively, even at higher concentrations.

### 3.2. Morphological Investigation

**Figure 7:**
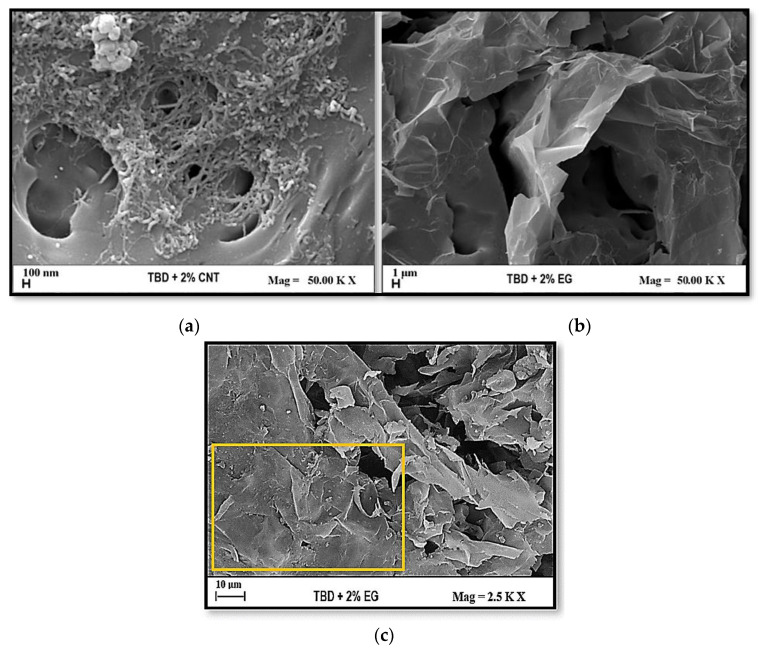
FE-SEM images of nanocomposites containing 2 wt% carbon nanotubes (MWCNTs) in panel (**a**) and 2 wt% exfoliated graphite (EG) in panel (**b**). FE-SEM images of nanocomposites containing 2 wt% exfoliated graphite, highlighting the tendency of the nanofillers to align with the graphene layers of the graphitic block in a direction parallel to the silicone mold base in panel (**c**).

The FE-SEM images in Figure 7 provide an essential insight into the morphology of nanocomposites containing 2% nanofillers: multiwalled carbon nanotubes (MWCNTs) are shown in panel (a) and exfoliated graphite (EG) are shown in panel (b), respectively. From the analysis of these figures, it is possible to observe folded graphene sheets resembling the draping of a textile with the parallel preferential orientation of graphene layers/blocks. In both cases, the dispersion of the nanofillers appears to be sufficiently achieved, which is crucial for optimizing the electrical, mechanical, and thermal properties of the composite. A conductive network that is well-structured guarantees appreciable benefits on the properties mentioned above, as already discussed and reported in our previous studies [38,39]. It was worth noting that, apart from the natural tendency of the graphitic layers to dispose of themselves in a parallel way, due to the π-π attractive interactions, the procedure for the sample preparations determined the preferential orientation of the graphitic layers. In fact, because of their large surface area and their interaction with the polymeric matrix, they tend to align with the graphene layer of the graphitic block in a direction parallel to the silicone mold base. This trend is particularly evident in Figure 7c, where it stands out more distinctly (see the yellow frame).

The morphology of the carbon nanotube-based composite shows an entangled network of CNTs embedded in the polymer matrix. This interconnected structure is beneficial for electrical conductivity as it facilitates charge transport by forming conductive pathways. From a thermal transport perspective, the highly conductive nature of individual CNTs (with intrinsic thermal conductivity exceeding 3000 W/mK) plays a dominant role. Heat conduction in the composite is expected to be more efficient within the plane of the sample, where CNTs establish continuous thermal pathways. However, in the through-plane direction, thermal conductivity enhancement is significantly lower. This limitation is attributed to the interfacial thermal resistance between the nanotubes and the polymer matrix, as well as phonon scattering at tube junctions and polymer intercalation regions. These factors hinder effective phonon transport across the composite thickness, reducing the improvement in thermal conductivity in the perpendicular direction. By contrast, the composite containing exfoliated graphite (EG) exhibits a characteristic layered morphology, where graphene sheets are dispersed but remain mostly parallel to the substrate. This structural arrangement has a profound impact on the thermal properties of the material. Due to the strong in-plane sp^2^ carbon–carbon bonds within graphene layers, thermal conductivity is extremely high along the planar direction, reaching values comparable to bulk graphite (above 5000 W/mK). However, in the through-plane direction, heat transport is significantly hindered by the weak van der Waals interactions between stacked graphene sheets. These weak interlayer bonds introduce considerable thermal resistance, leading to poor vertical heat transfer. As a result, the composite exhibits highly anisotropic thermal behavior, favoring lateral heat dissipation while limiting heat conduction perpendicular to the plane. In brief, both nanocomposites demonstrate anisotropic thermal properties due to their respective fillers’ intrinsic characteristics and dispersion. While CNTs create a fibrous network that enhances in-plane thermal transport with minimal impact on the through-plane direction, exfoliated graphite forms layered structures that maximize in-plane conductivity while significantly restricting vertical heat transfer. The FE-SEM images support these findings by revealing the distribution, connectivity, and orientation of the nanofillers, which directly influence the macroscopic thermal performance of the composites. These results are particularly relevant for applications requiring efficient lateral heat dissipation, such as thermal interface materials, electronic cooling systems, and advanced heat management coatings. 

### 3.3. Simulation Results

This section presents multiphysics simulation results examining the influence of the number and orientation of graphene platelets within the matrix on the thermal properties of the resulting materials. The analysis focuses on heat flux, thermal conductivity, and the spatial-temporal temperature distribution, offering insights into the heat transfer mechanisms governing these composites.

#### 3.3.1. Case Study of a Single Graphene Platelet Parallel to the xy-Plane

**Figure 8:**
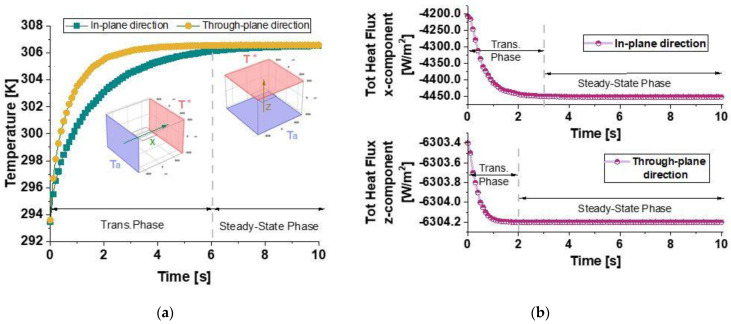
Temperature evolution over time for both investigated directions (in-plane and trough-plane) in panel (**a**) and the corresponding evolution of the total heat flux components along the in-plane (x-direction) and through-plane (z-direction) directions over time in panel (**b**).

The preliminary results presented in this subsection highlight the influence of a single graphene nanoplatelet (GNP) embedded in a resin matrix. The orientation of the GNP is set to 0 radians, meaning that it lies parallel to the x-y plane. Figure 8a presents the evolution of the temperature with time. The temperature decreases in both directions, but it is slower in the in-plane direction (x-direction) than in the through-plane direction (z-direction). Figure 8b illustrates the evolution of the total heat flux components with time along the in-plane (x-direction) and through-plane (z-direction) directions. Initially, during the transient phase, both flux components decayed sharply in accordance with the temperature decrease in the same directions, and then each component was shown to asymptote to a different constant value once the equilibrium conditions were reached. Specifically, the flux in the z-direction decayed faster than the x-direction and reached a steady-state value (Q_z_ = −6304.2 W/m^2^) after about 2 s, which is higher than the one reached along the x-direction after about 3 s (Q_x_ = −4451.6 W/m^2^) because the temperature gradient along z was much higher than the gradient in the x-direction, even though the thermal conductivity in the plane was higher than that across the plane.

On the other hand, considering the balance equations for each component in a single direction, it is possible to determine the characteristic time of the conductive phenomenon in a particular direction:

(11) $$\tau_{gj}=\frac{L_{igj}^{2}}{\alpha_{gj}}\;\;\;\;\;\;\;\;and\;\;\;\;\;\;\;\;\tau_{pj}=\frac{L_{ipj}^{2}}{\alpha_{pj}}\;$$

where α_gj_ and α_pj_ and L_igj_ and L_ipj_ represent the thermal diffusivity and length of the graphene and polymer subdomain in the j-direction. In particular, the characteristic times of the conductive phenomenon are longer along the x-direction for both the graphene and polymer considered separately so that the characteristic time of the overall conductive phenomenon in the entire composite domain appears slower in the x-direction than in the z-direction.

This implies that an increase in material length leads to a lower heat flux, as the heat requires more time to propagate through the material. This effect is more pronounced in directions where the characteristic conduction time is larger, such as in the *x*-direction. In brief, the heat flux decreases with increasing length, as a longer thermal pathway results in extended thermal equilibration times. The numerically calculated thermal conductivity of the resulting structure in the x-direction (λ_x_ = 0.2487 W/mK) is slightly higher than the intrinsic thermal conductivity of the pure resin (0.235 W/mK). This increase suggests that even a single graphene nanoplatelet enhances the in-plane heat transfer, albeit modestly. However, the thermal conductivity in the z-direction (λ_z_ = 0.2350 W/mK) remains almost identical to that of pure resin, indicating that the presence of the graphene platelet does not significantly impact heat transport in this direction. All numerically calculated thermal data are reported in Table 2.

**Figure 9:**
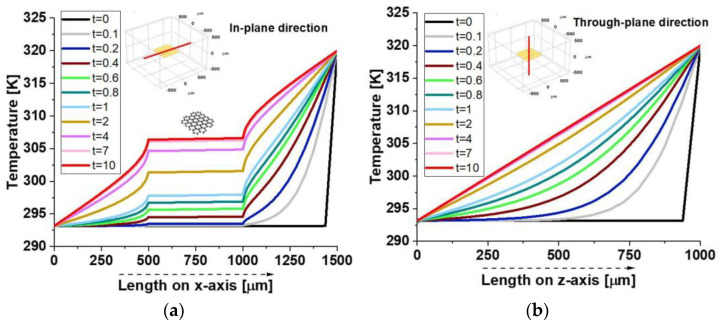
Temperature profiles along the symmetrical axes in both the in-plane (**a**) and through-plane directions (**b**) in in the case of a single graphene particle.

The two graphs in Figure 9 illustrate the temperature evolution along the symmetrical axes in both the in-plane direction in panel (a) and the through-plane direction in panel (b) of the composite material over time.

In the in-plane direction, the temperature distribution shows sharp discontinuities at approximately 500 µm and 1000 µm, corresponding to the resin–graphene platelet interfaces. Within the graphene region, the temperature remains constant, forming a plateau-like profile due to the platelet’s high thermal conductivity, whereas a gradual temperature gradient emerges in the surrounding resin. These discontinuities arise from the stark contrast in thermal properties, as the resin’s lower conductivity restricts heat conduction. Conversely, in the direction of the through plane, the temperature distribution follows a smooth gradient without significant jumps. The thin nature of the graphene platelet limits its influence on heat transfer, resulting in higher thermal resistance and less efficient conduction across its thickness compared to its plane. Overall, the results highlight the composite’s strong thermal anisotropy, with superior heat propagation along the graphene plane. This behavior is crucial for designing thermally conductive composites, particularly in applications like electronics, thermal management, and advanced structural materials.

**Figure 10:**
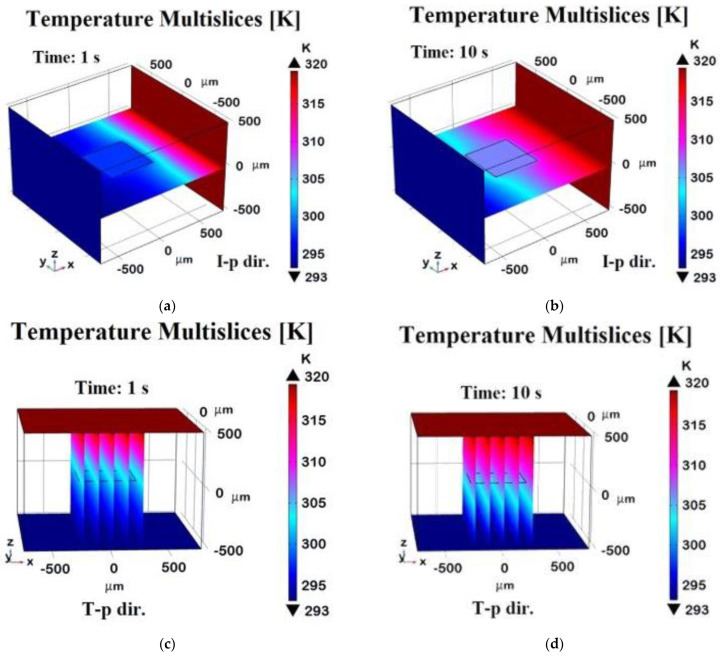
Temperature profiles along the in-plane direction (I-p dir.) at t = 1 s (transient phase, panel (**a**)) and t = 10 s (steady state, panel (**b**)) evaluated on a plane intersecting the graphene sheet. Panels (**c**,**d**) show the corresponding temperature distributions along the through-plane direction (T-p dir.) at the same time points computed on cross-sectional planes perpendicular to graphene’s xy-plane.

Figure 10 presents a 3D visualization of temperature distribution at t = 1 s and t = 10 s for in-plane (x-direction) and through-plane (z-direction) heat transfer. In detail, such temperature profiles are evaluated on a plane pass for the symmetric axis of the zy-plane distribution in the case of in-plane (I-p dir.) analysis or evaluated along transversal planes with respect to the xy-plane of graphene in the case of through-plane (T-p dir.) investigations. At t = 1 s in the x-direction (Figure 10a), a strong temperature gradient separates hotter and cooler regions, indicating incomplete heat diffusion. By t = 10 s (Figure 10b), the system reaches a near-steady state with a uniform thermal profile due to graphene’s high in-plane conductivity. Conversely, in the z-direction, temperature gradients remain more pronounced at t = 1 s (Figure 10c) and persist at t = 10 s (Figure 10d), though the gradient is somewhat smooth. This highlights graphene’s anisotropic thermal properties, with much lower conductivity in the through-plane direction.

**Figure 11:**
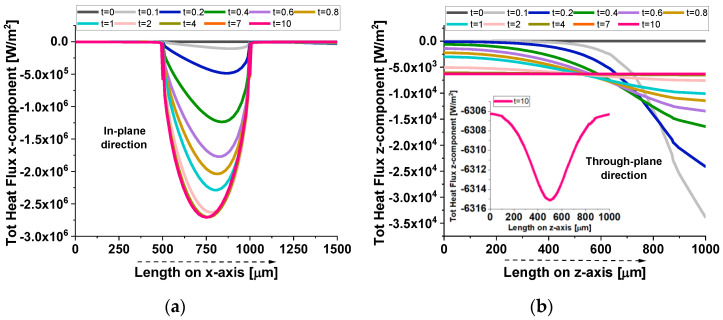
Total heat flux of the x-component evaluated in the in-plane direction (**a**) and the z-component evaluated in the through-plane direction (**b**) at different time instants in in the case of a single graphene particle.

Figure 11 presents the total heat flux, specifically the x-component for the in-plane direction (a) and the z-component for the through-plane direction (b), evaluated at different time points. In the in-plane direction (panel a), the graphene platelet spans the region from 500 µm to 1000 µm along the x-axis, occupying almost half of the observed length. The curves at different times (up to t = 10 s) reveal that the heat flux increases sharply within this interval, reflecting the high thermal conductivity of graphene compared to the surrounding resin. Over time, the flux profile evolves as heat penetrates the material, but the graphene region consistently exhibits a higher flux, indicating more efficient lateral heat transfer. In the through-plane direction (panel b), the platelet extends by only 1 nm, creating a much thinner conductive layer. The flux, therefore, rises in this narrow band, though less dramatically than in the in-plane case since the path for conduction is limited by the platelet’s small thickness. The inset in the right figure provides a magnified view of this zone, highlighting the local increase in flux as heat crosses the graphene layer. Despite the short distance, the platelet still enhances through-plane conduction relative to the surrounding resin, yet its impact is not as pronounced as it is along the in-plane axis.

**Figure 12:**
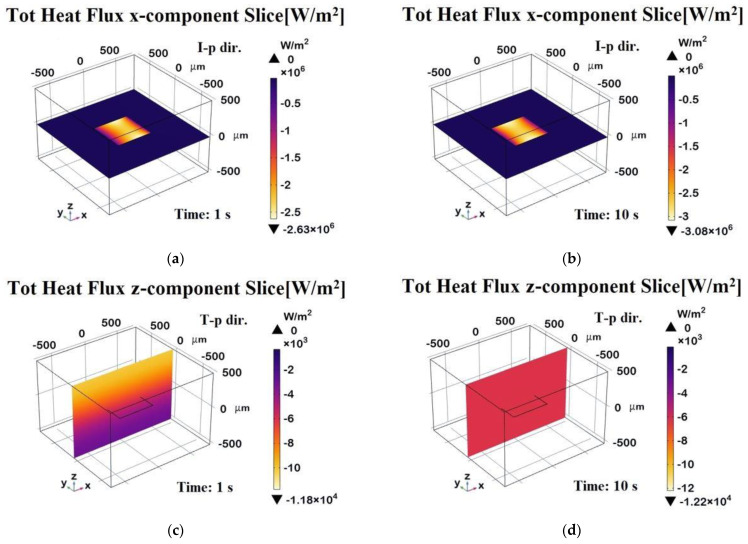
The total heat flux profiles along the in-plane direction (I-p dir.) at t = 1 s (transient phase, panel (**a**)) and t = 10 s (steady state, panel (**b**)) evaluated on a plane passing for the symmetric axis of the zy-plane and intersecting the graphene sheet. Panels (**c**,**d**) show the total heat flux distributions along the through-plane direction (T-p dir.) at the same time points computed on a transversal plane with respect to the graphene sheet. This plane passes for the symmetric axis of the xy-plane.

To conclude, the four 3D plots in Figure 12 illustrate the evolution of the heat flux component along different directions at different time points. The top row displays the heat flux in the **x-direction**, evaluated on a plane passing through the graphene sheet at t = 1 s (a) and at t = 10 s (b). The bottom row represents the heat flux distribution in the **z-direction**, evaluated along a transversal plane at t = 1 s (c) and at a steady state (t = 10 s, d). Compared to the previous 2D plots, these distributions provide insights into the temporal evolution and spatial localization of the heat transport within the domain. In the x-direction, at t = 1 s, the heat flux is concentrated near the graphene sheet, indicating strong in-plane conduction. By t = 10 s, the flux stabilizes at a higher value and spreads more evenly due to graphene’s high in-plane conductivity. In the z-direction, at t = 1 s, a strong gradient near the graphene sheet shows that heat is primarily dissipated through the plane. By t = 10 s, the distribution becomes more uniform.

The next few studies will explore how multiple graphene layers in a 3 × 3 arrangement with varied orientations influence overall thermal conductivity.

#### 3.3.2. Case Study—Influence of Multiple Graphene Platelets Orientation Relative to Heat Flux Direction on Thermal Properties

**Figure 13:**
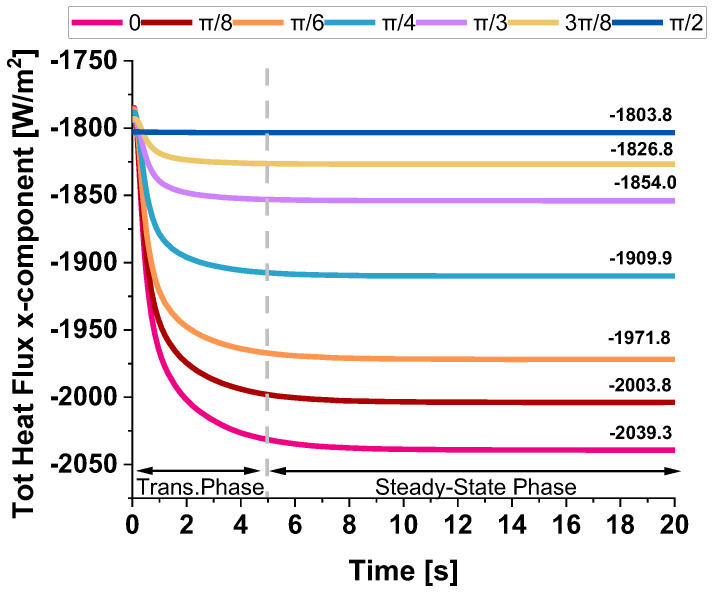
Evolution of the total heat flux, the x-component, over time for various angles (from 0 to π/2) between the heat flow direction and the graphene plane.

Given their highly anisotropic thermal conductivity, graphene platelets play a crucial role in determining heat transfer efficiency and the overall thermal performance of the material. Their orientation relative to the heat flux direction significantly influences the distribution and dissipation of thermal energy, affecting key properties such as thermal conductivity, heat flux pathways, and temperature gradients. Figure 13 illustrates the evolution of the total heat flux (x-component) evaluated in the in-plane direction over time for different angles between the heat flow and the graphene plane, specifically from 0 up to π/2. The observed behavior reveals two distinct phases. Initially, there is a rapid decrease in heat flux, indicating a transient phase where heat redistributes quickly within the system. This sharp decline suggests that the material promptly reacts to thermal excitation before stabilizing. As time progresses, the flux approaches a steady-state value after approximately 5 s, where the rate of change diminishes, and the system reaches equilibrium. The final flux values depend on the angle, with a clear trend showing that the heat flux decreases as the angle increases.

Table 3 provides a detailed analysis that underscores the significant anisotropy in the thermal transport properties of graphene, encapsulating the previously discussed heat flux values and the newly computed thermal conductivity in the x-direction for various graphene nanoplatelet (GNP) orientations. The data confirmed that graphene exhibits directional dependence in its thermal transport behavior, where heat transfer is most efficient when GNPs are aligned parallel to the heat flux direction. Specifically, at an orientation angle of 0°, the system demonstrates the highest thermal conductivity value of 0.2635 W/mK, accompanied by the highest heat flux of −2039.3 W/m^2^, highlighting graphene’s superior in-plane thermal transport properties. As the orientation angle increases, the thermal conductivity and heat flux progressively decrease. This reduction is more noticeable at intermediate angles, specifically at π/4 and π/3, where the values exhibit a more substantial decline, reflecting the inherent anisotropic nature of the material. At the extreme angle of π/2, which represents a perpendicular alignment to the heat flux direction, the thermal conductivity drops to 0.235 W/mK, and the heat flux reaches −1803.8 W/m^2^, confirming that a perpendicular alignment significantly impedes heat transfer, further illustrating the material’s anisotropic behavior. These results reinforce the concept that the efficiency of thermal conduction in graphene is highly dependent on its orientation relative to the heat flux direction. The findings also underscore the importance of considering this anisotropy when designing devices or materials that require precise control over thermal management, especially in applications where directional heat dissipation plays a critical role. The pronounced difference in thermal conductivity between the in-plane and through-plane directions reveals graphene’s potential in specific thermal management applications where heat needs to be directed or controlled in particular orientations.

**Figure 14:**
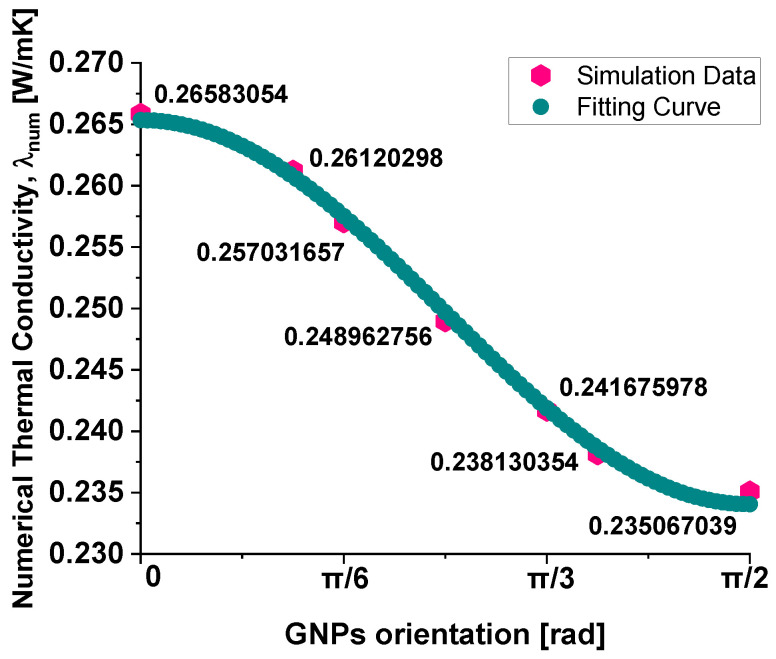
The fitting curve for determining an empirical law for the numerical thermal conductivity as a function of the GNPs’ orientation inside the polymeric host matrix.

The macroscopic thermal conductivity of the composite appears to follow the empirical law shown in Equation (13), which provides a good interpolation of the numerical data, as evident from the graphical plot in Figure 14.

(12) $$\lambda_{num}\left(\theta \right)=A-B{\cdot sin}^{2}\left(\theta \right)-C{\cdot cos}^{2}\left(\theta \right)$$

This equation expresses conductivity as a function of the sine and cosine squared terms, suggesting a dependency on the angular orientation of the graphene nanoplatelets, i.e.,$\;\lambda_{num}=f\left(\theta \right)$.

The optimal values for the coefficients A, B, and C, ensuring the best fitting results, are as follows: A = 0.07998, B = −0.15407, and C = 0.18536.

This correlation aligns with the assumption made for the intrinsic thermal conductivity of the individual graphene sheets ($\lambda_{g,\;intr})$, represented by the following equation:

(13) $$\lambda_{g,\;intr}\left(\theta \right)=\lambda_{i-p}{\cdot cos}^{2}\left(\theta \right)+\lambda_{t-p}{\cdot sin}^{2}\left(\theta \right)$$

The above equation models the thermal conductivity of anisotropic graphene, where the conductivity along different directions—denoted by θ, corresponding to the angle between the heat flow direction and the GNP plane—follows a power-law combination of cosine and sine terms.

This formula reflects the anisotropic behavior of graphene, which has the highest thermal conductivity when heat flows along the plane (θ = 0, so λ_g,intr_ (0) = λ_I-p dir._) and the lowest thermal conductivity when heat propagates perpendicular to the plane (θ = 90^∘^, so λ_g,intr_ (90°) = λ_T-p dir._).

By assuming such a dependence at the microscopic level for the individual GNPs, the macroscopic behavior of the composite naturally follows a similar angular dependency. The key idea is that the macroscopic conductivity results from the averaging of the anisotropic thermal properties of the individual graphene sheets, weighted by their orientation distribution within the composite. The interpolation law in the above Equation (13) thus emerges as a consequence of the orientation-dependent conductivity model used for the graphene inclusions.

#### 3.3.3. Case Study: Thermal Properties of Multiple Graphene Platelets (27) with Aligned, Random, and Perpendicular Orientations with Respect to the xy-Plane

**Figure 15:**
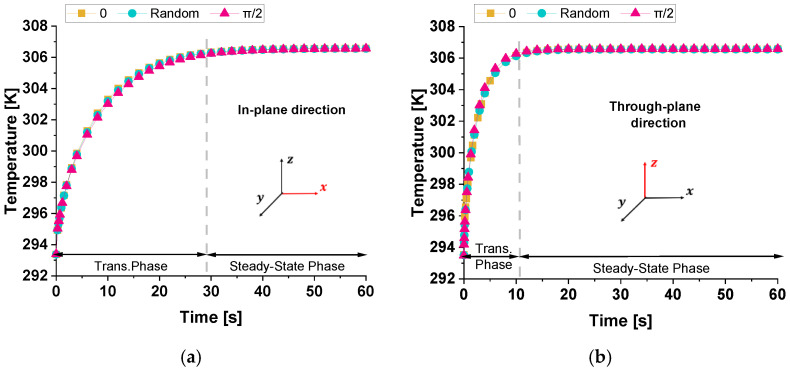
The evolution of temperature over the period (probe domain) when the temperature difference was applied along the in-plane direction in (**a**) and through-plane direction in (**b**) for different graphene nanoplatelet orientations (0, random, π/2) with respect to the *xy*-plane.

Figure 15 illustrates the time evolution of temperature (probe domain) for three different cases: 0, π/2, and the random orientations of graphene nanoplatelets relative to the xy-plane when the heat flow is in the in-plane direction (panel a) and in the through-plane direction (panel b). In both cases, since the variation in thermal conductivity among the three cases is relatively minor (λ_0_ ≈ λ_π/2_ ≈ λ_Random_), the heat transfer mechanism remains similar, leading to almost indistinguishable temperature responses. The comparison between the two graphs highlights a significantly slower transient in temperature evolution when the temperature difference is applied along the x-direction rather than the z-direction. This slower response (about 30 s against 10 s, respectively) is due to the greater spatial extent in the x-direction, which influences the heat diffusion dynamics. As theoretically expected, the equilibrium temperature remains unchanged.

**Figure 16:**
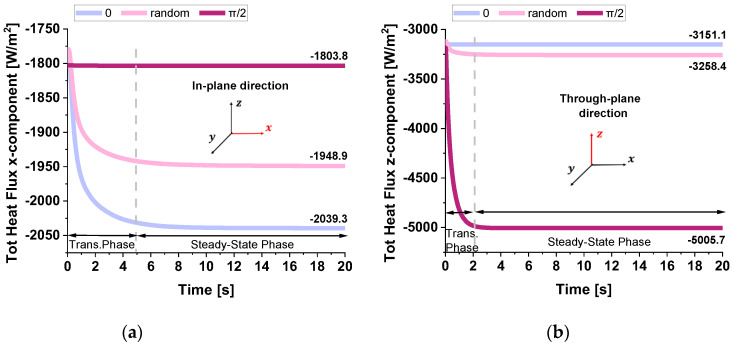
The total heat flux, x-component, computed in the in-plane direction (**a**), and the total heat, the flux, z-component, evaluated in the through-plane direction (**b**) vs. the time taken, up to 20 s, for different graphene nanoplatelet orientations (0, random, π/2).

The two plots in Figure 16 report the total heat flux (x-component) computed in the in-plane direction (panel a) and in the through-plane direction (panel b) for the three different GNPs orientations—0, random, and π/2—within the low-conductivity resin matrix.

Each curve exhibits a transient phase that transitions into a steady-state regime, although the transient period in the through-plane direction is notably shorter than in the in-plane direction (from about 3 s to about 5 s).

This difference arises because the distance over which the temperature difference that is applied in the z-direction is smaller, allowing the system to reach thermal equilibrium more rapidly.

The orientation of the GNPs, which have much higher thermal conductivity than the resin, plays a key role in determining the steady-state flux.

When GNPs are aligned parallel to the main heat flow (orientation 0), the effective thermal conductivity is higher, leading to a greater flux (−2039.3 W/m^2^).

Conversely, when they are perpendicular (π/2), the flux is lower (−1803.3 W/m^2^). In both directions, the random orientation yields intermediate results, reflecting a partial alignment effect.

These observations highlight the importance of anisotropy and the geometric configuration for achieving optimal thermal management in composite materials.

Once again, when comparing the transient phase durations across the temperature evolution (previous Figure 15) with those in the total heat flux, it is evident that the temperature transient phase is significantly longer. While the heat flux reaches steady-state conditions within a few seconds (approximately 5 s), the temperature requires around 10 or 30 s to stabilize. As previously discussed for a single graphene sheet, the prolonged transient temperature phase is due to the material’s thermal inertia, characterized by its heat capacity and diffusivity, while the rapid stabilization of the heat flux follows Fourier’s law, which depends directly on the imposed thermal gradient.

In summary, the presented numerical results suggest that in practical applications, modifying the orientation of graphene nanoplatelets within this range would not significantly affect the overall temperature field. However, it can still influence the local heat flux distribution, as different orientation values result in different steady-state heat fluxes.

Table 4 compiles the previously discussed heat flux data and the numerical thermal conductivities calculated in both analyzed directions for the different graphene sheet orientation scenarios. In the first configuration, where the graphene sheets are aligned at 0°, the corresponding thermal conductivity values are 0.2658 W/mK in the x-direction and 0.2351 W/mK in the z-direction. This configuration shows better heat transfer along the x-axis due to the alignment of the graphene sheets, but the z-direction conductivity remains relatively low. In the second configuration, the random orientation of GNPs results in more isotropic behavior, with slightly reduced thermal conductivity in the x-direction (0.2540 W/mK), which improves (0.2431 W/mK) in the z-direction compared to the 0° aligned case. In the third and last configuration, where the graphene sheets are aligned at 90°, the thermal conductivity values are 0.2351 W/mK in the x-direction and 0.3734 W/mK in the z-direction. This configuration greatly enhances heat transfer along the z-direction at the expense of reduced conductivity in the x-direction, demonstrating strong anisotropy. Overall, the 0° aligned configuration favors heat transfer in the x-direction, while the random configuration provides a balance between the x and z directions, making it more isotropic. In contrast, the *π*/2 aligned configuration enhances heat transfer in the z-direction, making it the best choice for applications requiring efficient vertical heat dissipation.

**Figure 17:**
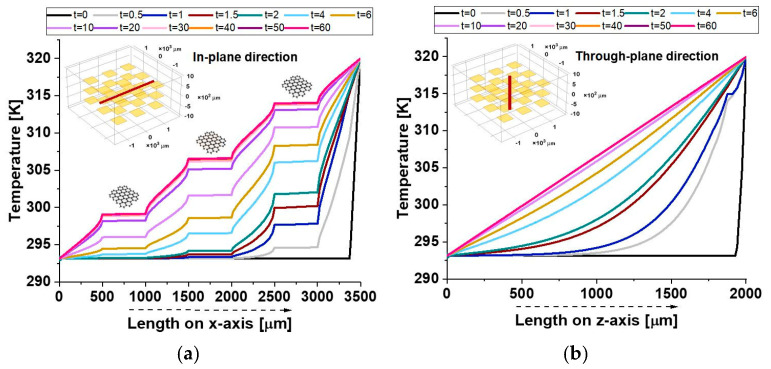
Temperature profiles along the symmetrical axes in both the in-plane (**a**) and through-plane directions (**b**) in in the case of 27 aligned graphene particles.

Figure 17 provides additional evidence of the composite’s anisotropic thermal response, as highlighted by the observed temperature distributions.

In the in-plane direction (a), plateaus correspond to the crossing of graphene nanoplatelets (GNPs), which, given their alignment along the analysis axis and their high intrinsic thermal conductivity, facilitate localized thermal equilibrium, reducing temperature gradients within their regions.

As a result, temperature gradients within these regions are minimized, allowing for efficient heat conduction along the plane.

On the other hand, the through-plane profile (b) exhibits a smooth temperature distribution, as the GNPs are only present in their minimal thickness direction. This limits their effectiveness in influencing the heat flow in the through-plane direction.

Consequently, the temperature remains more uniform in the through-plane profile, reflecting the dominance of lateral heat conduction within the composite material.

The relatively poor thermal conductivity in the through-plane direction can be attributed to weak interfacial thermal resistance and limited connectivity between the filler particles, which impede efficient heat transfer.

This stark contrast between the in-plane and through-plane profiles emphasizes the importance of the material’s orientation and structural properties in governing its thermal transport behavior.

**Figure 18:**
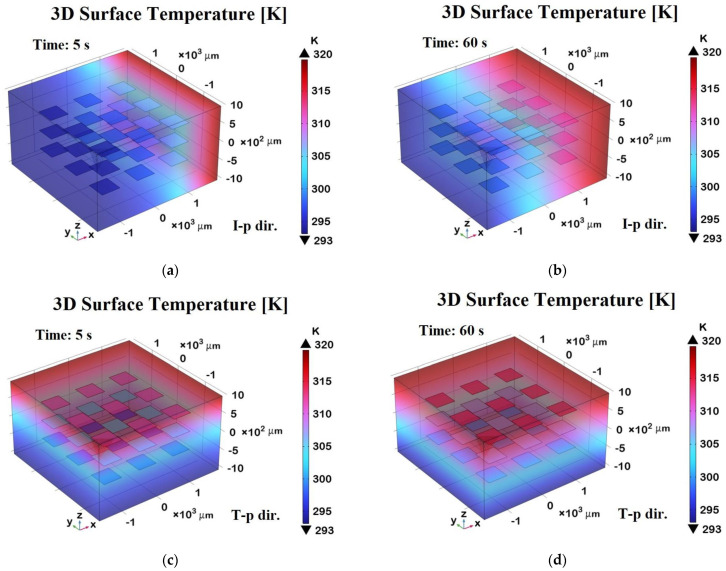
Three-dimensional surface temperature distributions along the in-plane direction (I-p dir.) at t = 5 s (transient phase, panel (**a**)) and t = 60 s (steady state, panel (**b**)). Panels (**c**) and (**d**) display the corresponding temperature profiles in the through-plane direction (T-p dir.) at t = 5 s and t = 60 s, respectively.

Figure 18 shows 3D surface temperature distributions at two different time points—t = 5 s (transient phase) and t = 60 s (steady state)—along two orthogonal directions relative to the graphene sheets embedded in the composite.

Panels (a) and (b) represent the in-plane (I-p dir.) temperature fields at 5 s and 60 s, respectively, while panels (c) and (d) show the through-plane (T-p dir.) distributions at the same time points.

In panel (a), during the transient regime, the temperature begins to diffuse primarily along the in-plane direction, following the high thermal conductivity pathways defined by the graphene sheets. After 60 s, as shown in panel (b), the temperature distribution becomes more uniform and widespread in-plane, indicating that the thermal equilibrium has been largely reached along this direction.

Panels (c) and (d), corresponding to the through-plane direction, clearly demonstrate a slower heat diffusion process due to the limited thermal conductivity of the resin matrix in the direction orthogonal to the graphene sheets. After 5 s (panel c), the heat penetration is still superficial, while at 60 s (panel d), a more gradual but still non-uniform distribution persists, highlighting the anisotropic thermal response of the composite.

Overall, the 3D representation in these panels offers a richer understanding of the heat propagation dynamics compared to the 2D plots, making the anisotropic effects and the role of graphene’s orientation more visually and quantitatively evident.

**Figure 19:**
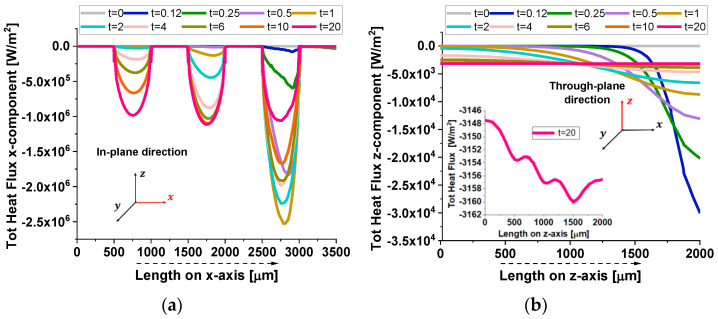
Total heat flux x-component evaluated in the in-plane direction (**a**) and z-component evaluated in the through-plane direction (**b**) at different time points in the case of 27 aligned graphene particles.

In Figure 19, the heat flux components further illustrate the anisotropic thermal transport in the composite.

In the in-plane direction (a), the flux exhibits periodic dips, aligning with the positions of graphene nanoplatelets (GNPs). This pattern results from the high thermal conductivity of GNPs, which locally redistribute heat, creating non-uniform flux variations. Conversely, in the through-plane direction (b), the flux decreases smoothly as heat encounters significant resistance due to the minimal thickness and weak interconnectivity of GNPs along this axis. Once again, this confirms that heat preferentially propagates along the in-plane direction, while through-plane conduction remains constrained by interfacial limitations.

**Figure 20:**
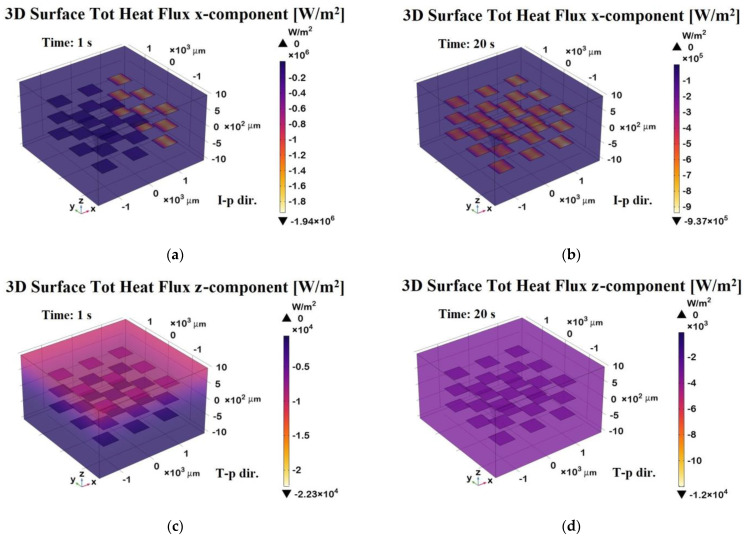
Three-dimensional surface distributions of the total heat flux. Panels (**a**,**b**) display the x-component along the in-plane direction (I-p dir.) at t = 1 s (transient phase) and t = 20 s (steady state), respectively. Panels (**c**,**d**) show the z-component of the total heat flux along the through-plane direction (T-p dir.) at the same time points.

The 3D surface heat flux distributions shown in Figure 20 complement the total heat flux previously discussed, providing further insight into the thermal behavior of the composite.

Panels (a) and (b) depict the x-component of the total heat flux (surface probe) in the in-plane direction (I-p dir.) at *t* = 1 s (transient phase) and *t* = 20 s (steady state), respectively. These plots highlight the preferential heat transport along the graphene sheets, which is consistent with their higher in-plane thermal conductivity. As a result, the heat flux is more highly concentrated in the graphene regions, which act as preferential thermal pathways, while the polymeric areas show lower thermal transport. Panels (c) and (d) illustrate the z-component of the heat flux in the through-plane direction (T-p dir.) at the same time points.

The magnitude of the flux in these panels is notably lower and more uniformly distributed compared to the in-plane direction, reflecting the limited thermal transport across the resin-dominated regions.

Overall, this figure emphasizes the strong anisotropic nature of the material, with heat preferentially propagating along the graphene-aligned in-plane pathways, which is especially evident in the early and steady-state phases. The periodic arrangement of the graphene plates suggests an engineered composite structure designed to enhance heat dissipation or directional thermal transport.

**Figure 21:**
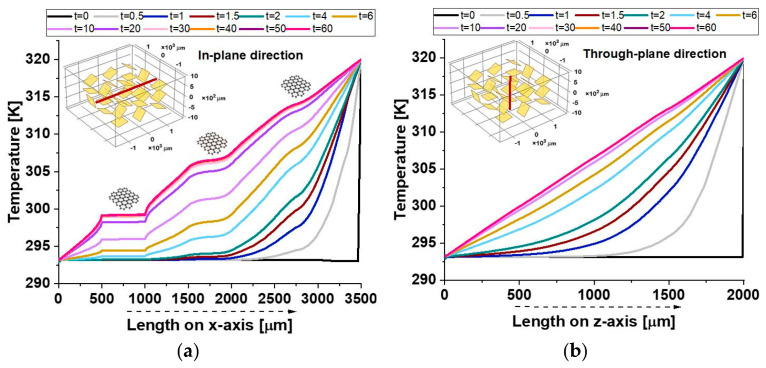
Temperature profiles along the symmetrical axes of the composite with a random distribution of 27 graphene platelets, as shown in the in-plane direction in panel (**a**) and in the through-plane direction in panel (**b**).

Figure 21 illustrates the thermal response of a composite material with randomly dispersed graphene platelets. Specifically, Figures (a) and (b) show the temperature profiles along the in-plane and through-plane directions, respectively, capturing the spatial distribution of heat. 

Contrary to the findings presented in the previously discussed results, in this case of randomly dispersed graphene platelets, which represents the most realistic scenario compared to an ordered arrangement of platelets, in the temperature profiles, a plateau is observed only when a high-thermal-conductivity graphene platelet, relative to the surrounding matrix, is entirely crossed by the cutline. This phenomenon corresponds to a parabolic shape in the heat flux profile. From a temporal perspective, temperature profiles remain briefly stable when the cutline transversely intersects the platelets, leading to the appearance of peaks in the heat flux graphs. These peaks arise due to the strong contrast in conductivity between the matrix and the platelets, causing rapid local variations in heat transport.

**Figure 22:**
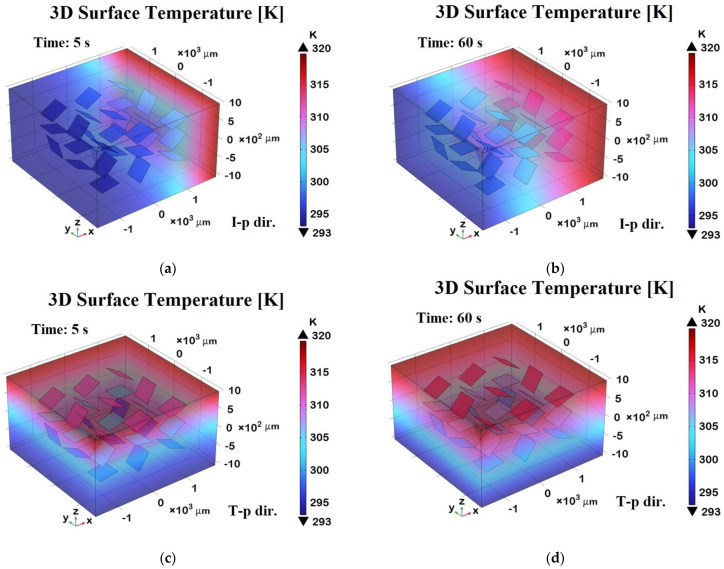
Three-dimensional surface temperature distributions in the case of randomly oriented GNPs in the in-plane direction (I-p dir.) at t = 5 s (transient phase, panel (**a**)) and t = 60 s (steady state, panel (**b**)). Panels (**c**) and (**d**) display the corresponding temperature profiles in the through-plane direction (T-p dir.) at t = 5 s and t = 60 s, respectively.

Figure 22 illustrates the 3D surface temperature distributions in a composite containing randomly oriented graphene nanoplatelets (GNPs), highlighting the impact of disordered alignment compared to the previously analyzed case with aligned platelets. Figure 22a and Figure 22b show the temperature profiles along the in-plane direction (I-p dir.) at t = 5 s (transient phase) and t = 60 s (steady state), respectively.

Compared to the aligned configuration, the temperature distribution here appears less uniform and more scattered, indicating reduced thermal conductivity in the in-plane direction. The heat spreads in a less directional and more diffused manner, underscoring the loss of thermal guidance provided by aligned platelets. Figure 22c,d display the corresponding temperature distributions in the through-plane direction (T-p dir.) at the same time points. A similar trend was observed, with less efficient heat transfer and more pronounced thermal gradients. The propagation of heat was visibly hindered, and the surface remained cooler relative to the aligned case, especially at early times. Overall, this figure clearly demonstrates that the random orientation of the GNPs weakens the material’s thermal anisotropy, leading to less effective and less directed heat transfer in both the in-plane and through-plane directions. The 3D visualizations are particularly effective in emphasizing these effects, making their contrast with the aligned configuration immediately apparent.

**Figure 23:**
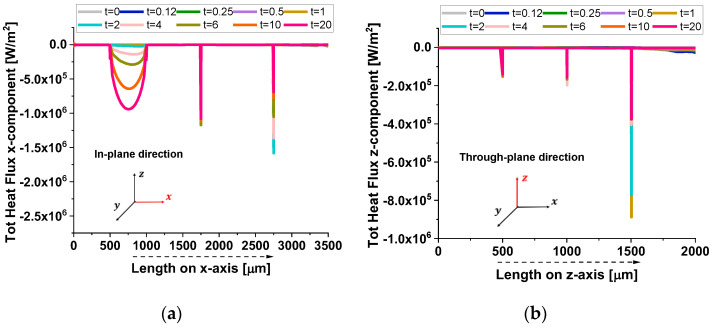
The total heat flux, the x-component, evaluated in the in-plane direction (**a**), and the z-component evaluated in the through-plane direction (**b**) at different time points in the case of 27 randomly oriented graphene particles.

The graphs in Figure 23 illustrate the total heat flux components in both the in-plane and through-plane directions at various time instants highlighting the flux variations assessed along the corresponding spatial cutlines. Distinct peaks in both components correspond to the regions where the heat flux intersects with graphene platelets, which are embedded in a low thermal conductivity resin. In the in-plane direction (Figure 23a), the first peak exhibits a complete bell-shaped profile, indicating that the heat flux fully traverses the first platelet. This suggests the favorable alignment of the platelet with respect to the x-direction, allowing uninterrupted conduction across its entire length. In contrast, the subsequent peaks are sharper and narrower, indicating only partial intersection with other platelets. These irregularities arise due to the random orientation of the graphene platelets within the resin. As a result, the heat flux encounters varying cross-sectional areas, leading to reduced and asymmetrical peak shapes. A similar behavior is observed in the through-plane direction (Figure 23b), where the z-component of the heat flux also displays localized peaks at positions where the flux path intersects the platelets. However, due to orientation and anisotropy, these peaks are generally less intense and more localized than those in the in-plane direction, reflecting the less efficient heat conduction across platelet thickness and through the resin matrix. These results clearly demonstrate the strong dependence of thermal transport behavior on the spatial orientation and distribution of high-conductivity inclusions within a composite material.

**Figure 24:**
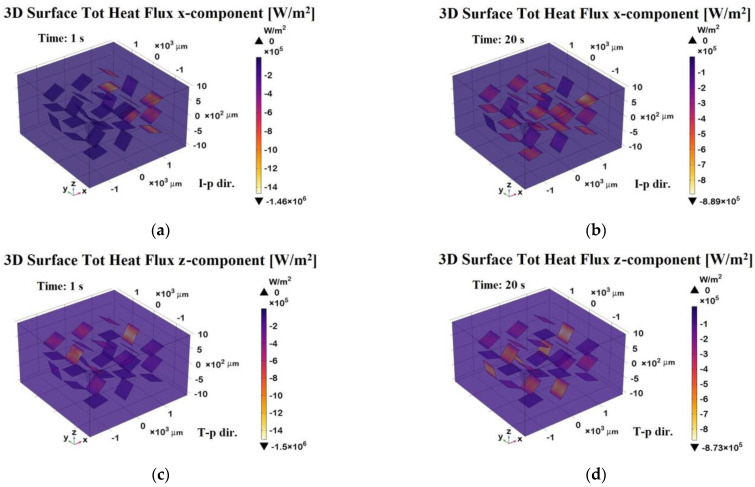
Three-dimensional surface distributions of the total heat flux in the case of randomly oriented GNPs within the resin. Panels (**a**,**b**) display the x-component along the in-plane direction (I-p dir.) at t = 1 s (transient phase) and t = 20 s (steady state), respectively. Panels (**c**,**d**) show the z-component of the total heat flux along the through-plane direction (T-p dir.) at the same time points.

The 3D surface distributions shown in Figure 24 complement the 2D results by providing spatial insights into the total heat flux behavior within the composite containing randomly oriented graphene nanoplatelets (GNPs). Panels (a) and (b) show the x-component (in-plane direction) of the heat flux at two time points: during the transient phase (t = 1 s) and at steady state (t = 20 s). Similarly, panels (c) and (d) depict the z-component (the through-plane direction) at the same time points. These 3D visualizations confirm and expand upon the findings from the 2D plots in Figure 23. Specifically, they illustrate how heat flux is locally intensified where the flux paths intersect with the platelets. Regions of high flux intensity (marked by peaks or valleys in the surface maps) correspond to areas where the GNPs are more directly aligned with the respective direction of heat flow. This is especially evident in the in-plane direction, where the anisotropic thermal conductivity of the platelets results in stronger and more continuous flux channels at the steady state. In the through-plane direction, the flux distribution appears more scattered and less intense, again reflecting the limited contribution of the GNPs in this orientation due to their thin geometry and poor alignment with the z-axis. Moreover, the differences between the transient and steady-state phases highlight how the redistribution of thermal gradients over time is influenced by the spatial arrangement and orientation of the platelets. Overall, this 3D analysis reinforces the conclusion that platelet orientation plays a critical role in governing thermal transport within the composite and provides a more comprehensive picture of heat flow patterns across all dimensions.

### 3.4. Theoretical Thermal Analysis

**Figure 25:**
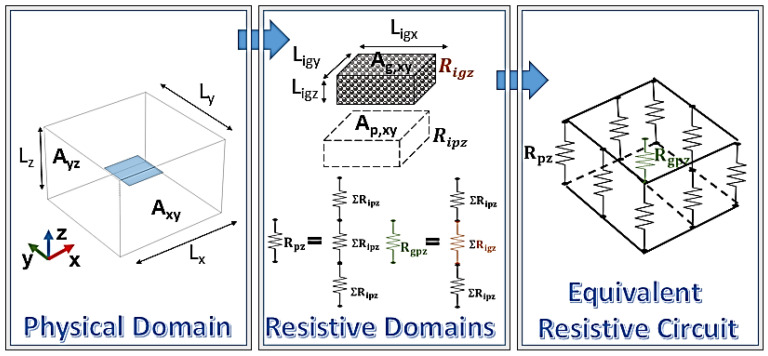
Thermal resistance network representation of a graphene-based structure.

The integration of thermal circuit theory was intentionally introduced to provide a complementary and mutually reinforcing interpretation of the thermal behavior of the nanocomposites. The thermal circuit model provides a simplified analytical perspective on anisotropic heat transfer, while COMSOL simulations deliver a detailed, three-dimensional analysis that captures the spatial effects of thermal property distributions. The added value of combining both approaches lies in their complementarity; while the thermal circuit model validates the physical consistency and theoretical grounding of the simulations, the simulations extend and contextualize the analytical results to more complex and realistic systems. Together, they build an interesting framework that not only explains the experimental observations but also supports the predictive capabilities of the model. Regardless of the number of graphene nanoplatelets and the observation direction, the methodology adopted for analyzing the thermal properties remains consistent. As illustrated in the simplest case of a single graphene sheet observed along the z-direction (Figure 25), the approach involves decomposing the physical domain into multiple resistive subdomains.

These subdomains are characterized by identical geometric parameters, with their lengths and cross-sectional areas matching those of the graphene structure in the direction of applied heat flow. The total resistance of the examined structure is determined by the series and parallel combination of various subdomain resistances, as schematically represented in Figure 25.

The primary factor that differentiates the analysis for a single graphene sheet from that of multiple nanoplatelets, or when considering alternative heat flow directions, is the number of resistive subdomains into which the system is partitioned. The corresponding resistive terms for the different analyzed cases are explicitly indicated below.

The provided Equation (16) describes the effective thermal resistances R_j_ in a hybrid graphene/polymer system along the j-direction:

(14) $$\frac{1}{R_{j}}=\alpha_{j}\frac{1}{R_{gpj}}+\beta_{j}\frac{1}{R_{pj}}\;with\;j=x\;or\;z$$

Such resistance, in each direction, is modeled as a combination of the thermal resistances associated with graphene–polymer hybrid domains (R_gpj_) and those of purely polymeric domains (R_pj_). The structural arrangement of these domains influences heat dissipation, similar to how electrical conductors in parallel distribute current. If the graphene-rich regions provide significantly lower thermal resistance compared to the polymeric regions, the heat flux will predominantly follow these pathways, leading to anisotropic thermal conductivity characteristics in the material.

The coefficients α_j_ and β_j_ likely represent weighting factors that account for the relative contribution of each domain to the overall thermal resistance. In detail, the coefficients α_j_ and β_j_ represent the amount of resistance in parallel on the surface orthogonal to direction j, consisting of various graphene–polymer series resistances and polymer-only series resistances, respectively.

This approach ensures that the polymeric contribution to thermal resistance is appropriately weighted according to the actual distribution of graphene platelets within the structure and consistently refers to the direction of exploration under analysis.

Each parallel branch, which may consist of either graphene/polymer hybrid subdomains or purely polymeric subdomains, is specifically evaluated according to the following relationships:

(15) $$R_{gpj}=\gamma_{j}\frac{L_{igj}}{\lambda_{gj}A_{g⊥j}}+\delta_{j}\frac{L_{ipj}}{\lambda_{p}A_{p⊥j}}=\frac{\gamma_{j}L_{igj}\left(\lambda_{p}A_{p⊥j}\right)+\delta_{j}L_{ipj}\left({K\lambda }_{gj}A_{g⊥j}\right)}{\left(\lambda_{gj}A_{g⊥j}\right)\left(\lambda_{p}A_{p⊥j}\right)}\;with\;j=x\;or\;z$$

The provided equation defines the hybrid thermal resistance *R_gpj_* in a series configuration for each parallel branch in the composite material, considering both the x- and z-directions, as indicated by the subscript j. This formulation is based on the second law of Ohm applied to heat conduction, which establishes that the total thermal resistance in a series arrangement is the sum of the individual resistances.

In this model, the resistance components are associated with graphene platelets and the polymer matrix, considering their distinct thermal conductivities and geometric characteristics. The term *L_igj_* represents the characteristic length of the graphene domain in direction *j*, while *L_ipj_* corresponds to the polymer subdomain. The thermal conductivity values *λ_gj_* and *λ_p_* describe the heat transport properties of graphene and polymer, respectively. It is important to note that graphene exhibits anisotropic thermal conductivity, meaning that *λ_gx_* ≠ *λ_gz_*, while the polymer is assumed to have isotropic conductivity *λ_p_*.

The parameters *γ_j_* and *δ_j_* account for the number of identical series resistances associated with graphene and polymer regions, respectively, within each parallel branch. Specifically, *γ_j_* quantifies the number of graphene platelets contributing to the series resistance along direction *j*, whereas *δ_j_* represents the number of polymer-only segments in the same direction. These factors are essential in accurately modeling the heat transfer pathways as they reflect the structural arrangement of the hybrid composite system. The given equation evaluates the thermal resistance *R_pj_* of purely polymeric parallel branches in the composite system, following the second law of Ohm for thermal conduction. This law states that for a series configuration, the total resistance is the sum of the individual resistances encountered along the heat flow path. In this formulation, the term *R_pj_* represents the overall resistance of the polymer subdomain along direction j, which, once again, can be either x or z. The expression accounts for the number of repeated polymeric resistances in the series by including the factor (γ_j_ + δ_j_) already defined above.

(16) $$R_{pj}=\left(\gamma_{j}+\delta_{j}\right)R_{ipj}=\left(\gamma_{j}+\delta_{j}\right)\frac{L_{ipj}}{\lambda_{p}A_{p⊥j}}\;with\;j=x\;or\;z$$

To conclude, Equation (17) estimates the heat flow Q_j_ along the direction j (where j = x or z) in accordance with Fourier’s law, as introduced in the previous Methods ssection and, more specifically, in Section 3.4 Thermal Circuity Theory. The expression accounts for the contributions of both the hybrid graphene/polymer and purely polymeric domains, weighted, respectively, by the different coefficients α_j_, β_j,_ γ_j_, and $\delta_{j}$. The temperature difference ΔT_j_ drives the heat flux, which is applied across the opposite interfaces of the representative volume in the considered direction. The given equation follows an analogy to Ohm’s law, where heat flow (Q_j_) behaves like electric current (I), temperature difference (ΔT_j_) resembles voltage (V), and thermal resistance (R_j_) corresponds to electrical resistance (R), reflecting Fourier’s law of heat conduction.

(17) $$Q_{j}=\frac{1}{R_{j}}∆T_{j}=\left[\alpha_{j}\frac{\left(\lambda_{gi}A_{g⊥j}\right)\left(\lambda_{p}A_{p⊥j}\right)}{\gamma_{j}L_{igj}\left(\lambda_{p}A_{p⊥j}\right)+\delta_{j}L_{ipj}\lambda_{gj}A_{g⊥j}}+\beta_{j}\frac{\left(\lambda_{p}A_{p⊥j}\right)}{\left(\gamma_{j}+\delta_{j}\right)L_{ipj}}\right]\cdot ∆T_{j}\;\;$$

This last equation provides a crucial component in modeling the effective thermal behavior of the hybrid graphene/polymer composite. By considering both series and parallel contributions of the composite’s microstructure, it ensures a comprehensive and precise representation of the material’s anisotropic heat transfer properties by enabling a detailed analysis of heat transport in different spatial directions. With reference to the above Equation (17), for a given temperature difference, higher thermal resistance leads to reduced heat flow because it impedes the transfer of thermal energy. Similarly, heat flux, which is the amount of heat transferred per unit area, is also influenced by thermal resistance. If R_j_ increases, the heat flux decreases as the material resists the flow of heat. Therefore, thermal resistance directly impacts thermal conductivity and, consequently, the heat flow and heat flux. Materials with lower thermal resistance offer higher thermal conductivity, resulting in higher heat flow and heat flux for a given temperature gradient. Conversely, materials with higher thermal resistance exhibit low thermal conductivity, which impedes heat transfer.

The values of all the aforementioned coefficients, evaluated in both the x and z directions and considering either single or multiple graphene platelets (27 in total), are systematically summarized in Table 5.

The following Table 6 presents all the computed values for the thermal parameters introduced by the thermal circuitry theory. The reported data include evaluations along the x and z-directions, considering both the simple case of a single graphene platelet and a more complex scenario involving multiple platelets (i.e., 27).

Table 7 compares the simulated and theoretical results for heat flux in both the through-plane direction (q_z_) and the in-plane direction (q_x_). The results show that the theoretical values match well with the simulated ones for both a single graphene nanoplatelet (GNP) and the 27-GNP configuration, exhibiting a negligible percentage difference, with a maximum deviation of 0.176% in the worst-case scenario. The agreement between the theoretical results and the outcomes of the numerical simulations validates the proposed 3D models, thereby also strengthening the interpretation of the experimental findings.

## 4. Conclusions

This study provides a comprehensive investigation into the anisotropic thermal conductivity of epoxy-based nanocomposites reinforced with low loadings of carbonaceous nanofillers, namely multiwall carbon nanotubes (MWCNTs) and exfoliated graphite (EG).

Rather than aiming to surpass the existing literature in terms of measured thermal conductivity values, this study provides a theoretical and numerical framework that supports and rationalizes the experimentally observed anisotropy, offering a complementary and mechanistic understanding of thermal transport in carbon-filled epoxy composites.

The experimental results obtained via the Transient Plane Source (TPS) technique highlight the critical role of filler geometry in dictating heat transport behavior within the polymer matrix. Specifically, MWCNTs were found to selectively enhance in-plane thermal conductivity with minimal effect in the through-plane direction, while EG nanoparticles produced significant conductivity gains in both directions, with a stronger influence along the plane. A key strength of this work lies in its integrated approach, where experimental observations are robustly supported by both multiphysics simulations and theoretical modeling. Three-dimensional simulations conducted in COMSOL Multiphysics validated and enriched the experimental findings by visualizing local heat flux distributions and confirming the directional nature of thermal transport induced by the nanofillers. The ability to couple numerical modeling with experimental data proved essential for justifying the measured thermal behavior and for predicting trends beyond the experimental range. Complementarily, the introduction of an analytical framework based on the thermal circuit theory allowed for a deeper interpretation of the mechanisms underlying anisotropic heat transfer in these heterogeneous systems. This model provided essential insight into how the spatial arrangement and orientation of the nanofillers affect the formation of conductive pathways.

Thermal conductivity was experimentally measured using the Transient Plane Source (TPS) technique, which enables the reliable evaluation of bulk thermal properties by analyzing the transient temperature response to a planar heat pulse. In the numerical simulations, thermal conductivity was evaluated based on Fourier’s law (see equation 10), where the heat flux q_j_ is related to the temperature gradient along direction j (with j = x or z) through the corresponding thermal conductivity λ_j_.

Although the theoretical and experimental results are not directly comparable in quantitative terms due to the idealized assumptions and simplifications in the theoretical and numerical models, both approaches are used to support and justify the experimentally observed trends, particularly in relation to the directions of investigation, x or z, of the material. The agreement between the theoretical predictions and experimental observations provides a qualitative validation of the results, supporting the proposed mechanisms behind the experimental findings. Overall, the synergy between experimental techniques, theoretical formulations, and computational modeling not only explains the observed anisotropic conductivity but also offers a reliable strategy for the rational design of thermally conductive polymer composites. The findings of this study have significant implications for various applications where efficient thermal management is crucial. It emerged that graphene-based nanocomposites, with their superior in-plane thermal conductivity, can be particularly beneficial in electronic devices and heat dissipation systems. Conversely, CNT-based composites, while more limited in their thermal conductivity, may still be advantageous in applications where mechanical strength and thermal stability are prioritized. These insights can guide the design of nanocomposite materials for a wide range of industrial applications, including thermal interface materials, batteries, and electronic packaging. To avoid making the current manuscript overly extensive, a future study will focus exclusively on the theoretical and numerical investigation of nanotube-based composites, allowing for a more in-depth analysis of their thermal behavior.

In summary, this study provides a significant contribution to the scientific community by offering an integrated experimental, theoretical, and numerical framework to better understand the anisotropic thermal conductivity of epoxy-based nanocomposites. While anisotropic behavior has been extensively discussed in the existing literature, our work is one of the first to employ both the thermal circuit theory and COMSOL simulations to systematically elucidate the underlying mechanisms. This approach not only enhances our understanding of heat transport phenomena in these materials but also provides a predictive tool for the design and optimization of composite systems with improved thermal properties, which is essential for advancing high-performance thermal management technologies.

## Figures and Tables

**Table 1:** Initial and boundary conditions for solving the thermal energy Equations (1) and (2).

Initial (I.C.) and Boundary (B.C.) Conditions *		Condition	Validity
I.C.	t = 0	T = Room Temperature (T_0_)	∀x,∀y,∀z
B.C.	i−p direction x = 0 t−p direction *z = 0*	T=293.15 K T=293.15 K	$\left(\forall y,\forall z,t>0\right)$ (∀x,∀y,t>0)
B.C.	i−p direction x = Lx t−p direction z=Lz	T=320 K T=320 K	(∀y,∀z,t>0) (∀x,∀z,t>0)

* All other external surfaces, if not subjected to imposed temperatures, were considered adiabatic, while the continuity condition was applied to all internal contact surfaces between different materials.

**Table 2:** Thermal properties evaluated for the case study of a single graphene platelet parallel to the x-y plane.

Configuration	GNPs Orientation [rad]	Heat Flux x-Direction q_x_ [W/m^2^] Thermal Conductivity λ_x_ [W/mK]	Heat Flux z-Direction q_z_ [W/m^2^] Thermal Conductivity λ_z_ [W/mK]
* 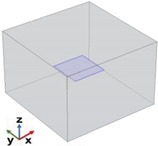 *	0	−4451.6 0.2486927	−6304.2 0.2350281

**Table 3:** Thermal properties evaluated for the case study of multiple graphene platelet counts (27) arranged in different fixed orientations with respect to the x-y plane.

**Configuration of** **GNPs’ Orientation [rad]**	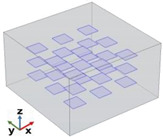 0	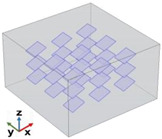 π/8	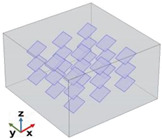 π/6	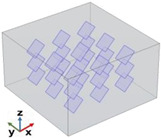 π/4
**Heat Flux q_x_ [W/m^2^] ** **Thermal Conductivity λ_x_ [W/mK]**	−2039.30 0.2658305	−2003.80 0.2612029	−1971.80 0.2570317	−1909.90 0.2489628
**Configuration of** **GNPs’ Orientation [rad]**	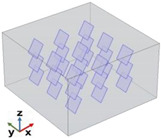 π/3	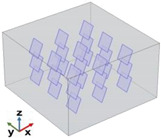 3π/8	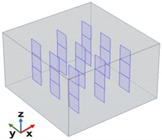 π/2	
**Heat Flux q_x_ [W/m^2^] ** **Thermal Conductivity λ_x_ [W/mK]**	−1854.00 0.2416759	−1826.80 0.2381304	−1803.30 0.2350670	

**Table 4:** Thermal properties evaluated for the case study of multiple graphene platelet counts (27) arranged in 3 different orientations with respect to the x-y plane, i.e., aligned (0), random, and transverse (*π/2*).

**Configuration of** **GNPs’ Orientation [rad]**	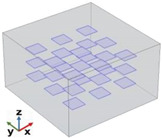 0	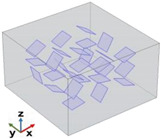 Random	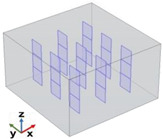 π/2
**Heat Flux in x-Direction q_x_ [W/m^2^]** **Thermal Conductivity λx [W/mK]**	−2039.3 0.2658305	−1948.9 0.2540466	−1803.3 0.2350670
**Heat Flux in z-Direction q_z_ [W/m^2^]** **Thermal Conductivity λz [W/mK]**	−3151.1 0.2350709	−3258.4 0.2430754	−5005.7 0.3734234

**Table 5:** Thermal subdomain properties within the discretized physical domain.

Configuration:	α_j_	β_j_	γ_j_	δ_j_
1 GNP z-direction	1	8	1	10^+6^–1
1 GNP x-direction	1	3 × 10^+6^–1	1	2
27 GNPs z-direction	9	40	3	3 × 10^+6^–3
27 GNPs x-direction	9	14 × 10^+6^–9	3	4

**Table 6:** Calculated values for the thermal parameters based on the presented thermal circuit theory.

Property/Sample	1 GNP x-Direction	1 GNP z-Direction	27 GNPs x-Direction	27 GNPs z-Direction
**Total Thermal Resistance ** **of Graphene/Polymer [W]**	R_gpx_ = 8.036 × 10^+9^	R_gpz_ = 1.702 × 10^+4^	R_gpx_ = 1.502 × 10^+10^	R_gpz_ = 3.404 × 10^+4^
**Total Thermal Resistance ** **of Polymer [K/W]**	R_px_ = 1.205 × 10^+10^	R_pz_ = 1.702 × 10^+4^	R_px_ = 2.629 × 10^+10^	R_pz_ = 3.404 × 10^+4^
**Total Thermal Conductance** **of Structure [W/K]**	1/R_x_ = 2.489 × 10^−4^	1/R_z_ = 5.288 × 10^−4^	1/R_x_ = 5.326 × 10^−4^	1/R_z_ = 1.439 × 10^−3^
**Heat Flow ** **Q [W]**	Q_x_ = −6.683 × 10^−3^	Q_z_ = −1.420 × 10^−2^	Q_x_ = −1.430 × 10^−2^	Q_z_ = −3.865 × 10^−2^
**Heat Flux ** **q [W/m^2^]**	q_x_ = −4455.31	q_z_ = −6309.75	q_x_ = −2042.90	q_z_ = −3154.87

**Table 7:** Comparison between simulated and theoretical results, including the relative error percentage (% Change), for the heat flux in the trough-plane direction (q_z_) and in-plane direction (q_x_).

Configuration:	q_z_ Simul. [W/m^2^]	q_z_ Theoret. [W/m^2^]	% Change	q_x_ Simul. [W/m^2^]	q_x_ Theoret. [W/m^2^]	% Change
1 GNP	−6304.02	−6309.75	0.091	−4451.60	−4455.31	0.083
27 GNPs	−3151.10	−3154.87	0.119	−2039.30	−2042.90	0.176

## Data Availability

All the necessary data are included in the text.
